# An Imaged Capillary Isoelectric Focusing Separation of the Linear and Cyclic Variants of a Mimotope of the Cancer‐Related CD20 Antigen–Validation and Statistical Evaluation

**DOI:** 10.1002/jssc.70054

**Published:** 2024-12-30

**Authors:** Georg Bloderer, Luigi Grassi, Chiara Cabrele, Hanno Stutz

**Affiliations:** ^1^ Department of Biosciences and Medical Biology University of Salzburg Salzburg Austria

**Keywords:** cyclic and linear peptide, iCIEF, mimotope, statistical evaluation, validation

## Abstract

Imaged capillary isoelectric focusing was successfully applied for separating an in‐house synthesized closely related peptide pair, that is, a linear 12‐mer (Rp5‐L) and its cyclic 15‐mer variant (Rp5‐C). Rp5‐L represents a mimotope, that is, an epitope mimicking peptide, of the CD20 antigen, which is over‐expressed in B‐cell‐related tumors. Peptide identity—including the successful disulfide bond formation in Rp5‐C—was confirmed with matrix‐assisted laser desorption ionization‐time of flight mass spectrometry. The purity of synthesized products was determined by a reversed‐phase high‐performance liquid chromatographic method with ultraviolet detection. The apparent isoelectric point (p*I*) of cyclic Rp5‐C and Rp5‐L was 5.99 and 6.47, respectively. An appropriate combination of carrier ampholytes allowed for their baseline separation with an analysis time of <20 min. Method validation was done for the synthesized peptides and three flanking p*I* markers covering, for example, repeatability and intermediate precision. Calibrations on different days resulted in identical slopes for Rp5‐L and Rp5‐C, respectively, as statistically confirmed by Welch's *t*‐test and pooled *t*‐test over 8 days. The calibration data of mimotopes and p*I* markers were evaluated for outliers, normality, homoscedasticity, and autocorrelation with complementary statistical procedures, which identified an otherwise unnoticed outlier for a p*I* marker. The linearity of calibration for Rp5‐L, Rp5‐C, and the p*I* markers was tested with Mandel's fitting test and lack‐of‐fit test. For Rp5‐L and Rp5‐C, the calculated limits of detection and limits of quantification were ≤0.31 and ≤0.96 µmol/L, respectively.

**Abbreviations**
CA: carrier ampholyte
CDR: complementary‐determining region
CI: confidence interval
CV: coefficient of variation
DF: degrees of freedom
DFBETAS: standardized difference in fits of beta
DFFITS: standardized difference in fit
DW: Durban‐Watson
ICH: International Council for Harmonization of Technical Requirements for Pharmaceuticals for Human Use
iCIEF: imaged capillary isoelectric focusing
LOD: limit of detection
LOF: lack‐of‐fit
LOQ: limit of quantification
mAb: monoclonal antibody
MALDI‐TOF‐MS: matrix‐assisted laser desorption ionization‐time of flight mass spectrometry
MC: methylcellulose
MFT: Mandel's fitting test
OLSM: ordinary least square method
PL: Pharmalyte
Q‐Q: quantile‐quantile
Rp5‐C: cyclic variant of the linear CD20 mimotope Rp5‐L
Rp5‐L: linear mimotope of CD20
RP‐HPLC‐UV: reversed‐phase high‐performance liquid chromatographic method with ultraviolet detection
R_S_
: electrophoretic resolution
SDR: studentized deleted residual
SE: standard error
SW: Shapiro‐Wilk
WCID: whole column image detection

## 1 Introduction

Antigen‐antibody interactions are based on the recognition of a specific epitope by the complementary‐determining region (CDR) of an antibody [1]. Thereby, an epitope represents a three‐dimensional topography on the protein surface formed by defined amino acids in the recognized domain [2, 3]. The interaction is rather on the atomic than on the residue level [4] and is governed by electrostatic and hydrophobic forces [3, 5]. Protein regions recognized by CDRs of monoclonal antibodies (mAbs) are either (i) linear or (ii) arranged spatially apart and brought to the vicinity by the antigen conformation. Thus, epitopes are classified as (i) continuous or (ii) discontinuous [2, 4, 6, 7]. Mimotopes are peptide surrogate immunogens, which mimic a genuine antigen epitope by imitating its antigenic activity [8]. However, the mimotope and its natural epitope do not necessarily share a consensus sequence or a related structure [4, 9]. Mimotopes offer versatile applications in diagnostics and therapy and can additionally support the standardization of therapeutic mAbs. Mimotopes proved their potential as diagnostic tools by recognizing autoantibodies as biomarkers [10], as vaccines in cancer immunotherapy [11, 12, 13], and in the treatment of allergic disorders [13, 14, 15] and neurodegenerative diseases [16, 17]. To ensure product safety and efficacy of mAbs, which are the top sellers of the biopharmaceutical industry [18, 19], their compliance with distinct specifications, that is, related acceptance criteria that define the product quality, is requested. This comprises defined properties [20] including the critical quality attribute of CDR integrity [21]. CDR consistency can be confirmed by standardized binding studies involving (model) antigens with well‐defined epitopes [8, 20, 22]. For this purpose, besides the intact genuine antigen that carries the target epitope [23, 24] mimotopes offer a valuable and economic alternative [25, 26]. Indeed, mimotopes are generally smaller than native epitopes, and can thus be chemically synthesized on a large scale with defined quality, lack infectious contaminations, and can be stored for extended periods of time [12].

Rituximab is a therapeutic mAb in the treatment of non‐Hodgkin lymphomas, rheumatoid arthritis, and neurodegenerative diseases [27]. It recognizes CD20, a transmembrane‐phosphoprotein expressed on B‐lymphocytes, as a target antigen [27, 28] and clears malignant B‐cells by induced apoptosis or complement‐mediated cytotoxicity [29]. The discontinuous epitope of CD20 is formed by a cyclic structure due to a disulfide bond and is situated in an extracellular loop of the protein [9, 30]. From the different mimotopes published for CD20 [31, 32, 33, 34], a linear mimotope—previously assigned as Rp5‐L ‐was selected [32, 33] and complemented by its cyclic pendant (Rp5‐C). The advantages of cyclic mimotopes are outlined elsewhere [12, 35]. Both peptides contain the same consensus sequence WPxWLE, which is recognized by rituximab [32, 34].

Capillary isoelectric focusing (CIEF) and progressively imaged CIEF (iCIEF) are employed in the charge profiling of biopharmaceutical products and biomolecules, comprising mRNA vaccines [36], virus capsid proteins [37] and especially mAbs [38, 39, 40, 41, 42]. Recently, a comparison of CIEF and iCIEF [43] as well as advanced iCIEF methods, such as digestion‐assisted iCIEF [44] and iCIEF‐MS [45], have been applied in the characterization of therapeutic mAbs. Due to the increased UV absorption of carrier ampholytes (CAs) at 200 nm‐230 nm, which refers to the wavelength interval where peptide bonds absorb, detection at 280 nm is required [46, 47]. Recent reviews surveyed capillary electrophoresis‐based separation methods applied in the analysis and characterization of peptides with particular emphasis on therapeutic peptides [47, 48]. The latter constitute biopharmaceuticals of emerging relevance [48]. However, (i)CIEF characterization of peptides is less common [48, 49] since at least one aromatic amino acid is required in the primary sequence [50]. The principles of (i)CIEF were outlined in numerous reviews [50, 51, 52]. Contrary to conventional CIEF, a mobilization of focused analyte zones is not required in iCIEF, and real‐time monitoring of the separation progress helps to prevent premature discontinuation of the focusing step [53, 54]. Moreover, a disruption of the formed pH gradient [53] with concomitant losses in resolution, which are both due to mobilization, are avoided [55], and the plateau effect, pH gradient drifts [56] as well as precipitation artifacts by over‐focusing [57] are prevented or better recognized.

For the first time, iCIEF is applied herein for the analysis and quantification of closely related mimotopes, Rp5‐L and Rp5‐C, which possess different conformations but presumably highly similar isoelectric points (p*I*s). Commercial p*I* markers were included for p*I* determination and as model compounds. Validation of the iCIEF method was done according to the guideline Q2(R2) of the International Council for Harmonization of Technical Requirements for Pharmaceuticals for Human Use (ICH) to demonstrate its applicability [58]. Usually, published validations lack the essential aspect of chaperoning statistical data analysis. The performed method validation in combination with advanced statistical measures allows for a comprehensive multi‐angle data evaluation, thus ensuring an improved insight into the data structure and an in‐depth elucidation of possible pitfalls. This validation aims to investigate iCIEF as a fast and cost‐effective tool in the evaluation of synthesized peptides/mimotopes orthogonal to other standard methods ideally providing an additional complementary analytical strategy [35, 59].

## 2 Materials and Methods

### 2.1 Imaged Capillary Electrophoresis System

iCIEF was done with a CEInfinite C01 system from Advanced Electrophoresis Systems Ltd. (AES Ltd., Cambridge, ON, Canada) using a whole column imaging detection (WCID) separation cartridge (from AES Ltd.) with 50 mm length, 200 µm id and 365 µm od. The WCID cartridge had an internal 0.1 µm fluorocarbon‐based coating and integrated electrolyte vessels for anolyte and catholyte solutions. Semi‐permeable membranes between the electrolyte vessels and the separation capillary assured electrical contact and allowed for a pass‐over of H_3_O^+^ and OH^−^ ions in the separation capillary in order to form the primary pH gradient. Analytes were detected at 280 nm with a deep UV‐LED over the entire length of the WCID cartridge using a complementary metal oxide semiconductor‐based detector array with images taken every 20 s.

### 2.2 Chemicals

Anolyte (80 mmol/L H_3_PO_4_ with 0.1% (m/v) methylcellulose (MC)) and catholyte (100 mmol/L NaOH with 0.1% (m/v) MC) solutions, 1% (m/v) MC, and marker compounds with p*I* 4.22, 4.65, 5.12 and 7.05 were kindly provided by AES Ltd. Carrier ampholytes AESlyte HR 3–10 and AESlyte SH 6–9, both with 35±5% (m/v), were from AES Ltd. Pharmalyte 3–10 (PL 3–10) and PL 5–6, both with 36% (m/v), were from GE Healthcare Bio‐Sciences (Waukesha, WI, USA). Ultrapure water Type 1 with a resistivity >18.0 MΩ·cm was provided by a Millipore Integral 3 system from Merck‐Millipore (Molsheim, France). Reagents and solvents for peptide synthesis and analysis are given in the Supporting Information.

### 2.3 Peptide Mimotopes

Based on the published mimotopes of CD20, a 12‐mer of the following sequence was selected: QDKLTQWPKWLE (Rp5‐L, linear mimotope of CD20) [32, 34]. In addition, a cyclic variant containing a disulfide bond was prepared, that is, CQDKLTQWPKWLEGC (Rp5‐C). Both peptides were synthesized in‐house. Further details together with the settings applied in their characterization with a reversed‐phase high‐performance liquid chromatographic method with ultraviolet detection (RP‐HPLC‐UV) and matrix‐assisted laser desorption ionization‐time of flight mass spectrometry (MALDI‐TOF‐MS), as well as the corresponding HPLC chromatograms and MALDI‐TOF‐MS spectra, are given in the Supporting Information (Figures S1–S3).

### 2.4 Sample Preparation for iCIEF

The commercial p*I* marker solutions (p*I* 4.65, 5.12, and 7.05) were diluted 1:10 (v/v) with ultrapure water. These dilutions were used in the preparation of iCIEF samples. The different sample compositions that were used in the iCIEF optimization are specified in section 3.1.1. Samples applied in the validation experiments contained 0.35% (m/v) MC, 0.50% (m/v) PL 3–10, 1.0% (m/v) PL 5–6, Rp5‐L, Rp5‐C, and the p*I* markers with concentrations specified in the respective sections. In their preparation, iCIEF samples were mixed and centrifuged with a mini‐centrifuge three times alternately. Finally, samples were centrifuged at 18 600 × *g* and 20°C for 5 min to eliminate air bubbles and particulate matter. All samples were prepared immediately prior to their injection.

### 2.5 Imaged CIEF

WCID cartridges were rinsed by means of a syringe with 0.35% (m/v) MC in ultrapure water for 5.0 min. Sample injection was done for 2.0 min thus entirely filling the separation cartridge. A profile scan over the imaging cartridge with intensity values between 8000 and 15 550 absorbance units ensured appropriate iCIEF operation. Focusing of analytes was done in three steps to limit the current to <20 µA: 500 V for 1.00 min, followed by 1000 V for 1.00 min, and finally 3000 V for 16.00 min. Anolyte and catholyte solutions were replenished every 10 runs. Prior to their storage overnight or over the weekend, WCID cartridges were rinsed with 0.35% (m/v) MC in ultrapure water for 5.0 min followed by a rinsing step with ultrapure water for 2.0 min. Finally, 0.5 mL air was injected into the cartridge to ensure its storage in a dry state. Prior to new iCIEF runs, cartridges were rinsed again with 0.35% (m/v) MC in ultrapure water for 5.0 min after dry storage.

### 2.6 Software and Data Processing

Data acquisition was done with CEInfinite C01 software (from AES Ltd.). For signal integration, data files in pixel format were either directly imported or first transformed from the original pixel scale to a p*I* scale before electropherograms were imported into Chromeleon 7 (v7.2.3.7553) from Thermo Scientific Dionex (Sunnyvale, CA, USA) and analyzed by the integration tool Cobra Wizard. Statistical data evaluation was done by IBM SPSS Inc. statistical software package version 29 (Chicago, Il, USA). Results were considered significant if *p *< 0.05 unless stated otherwise. Mandel's fitting test (MFT), lack‐of‐fit (LOF) test [60], the comparison of calibration slopes via Welch's *t*‐test and pooled *t*‐test as well as the calculation of the degrees of freedom (DF) according to the Aspin‐Welch approach [61] was done in Excel 365 (Microsoft; Redmond, WA, USA). The results of the LOF test were cross‐checked with SPSS. Figures were prepared by means of SPSS, SigmaPlot 15 from Inpixon (Palo Alto, CA, USA), and Excel.

## 3 Results and Discussion

Physicochemical properties of Rp5‐L and Rp5‐C relevant for iCIEF are given in Table S1. The mimotope consensus motif is WPxWLE (see section 2.3) [32, 33, 34]. Both Rp5‐L and Rp5‐C shared the same primary sequence. Rp5‐C had additional flanking residues that were introduced to obtain a cyclic variant of Rp5‐L by disulfide bond formation (see section 2.3; Table S1). The disulfide bond between the flanking Cys residues was formed by air oxidation and was confirmed by MALDI‐TOF‐MS data (Figure S2).

### 3.1 iCIEF Separation

#### 3.1.1 Separation Optimization

The similarity of Rp5‐L and Rp5‐C allows for testing the capability of iCIEF in separating different peptide conformations. According to an in silico computation with two different algorithms, based on their primary sequence both peptides shared the approximate same p*I* calculated with 6.05 and 6.07 (by *Expasy;* https://web.expasy.org/compute_pi/) or 6.25 for both (by *Protcalc;* https://protcalc.sourceforge.net/). This p*I* equality reflects the general assumption of both algorithms, that oligopeptides adopt linear structures. A third algorithm, that is, the *Isoelectric* *Point* *C* *alculator* (IPC; http://isoelectric.org/calculate.php), predicted a difference of 0.68 p*I* units, that is, 5.88 (Rp5‐C) and 6.56 (Rp5‐L) [62] (Table S1).

**FIGURE 1:**
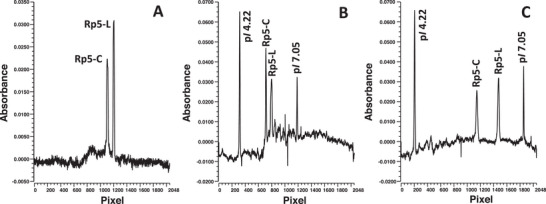
Optimization of the imaged capillary isoelectric focusing (iCIEF) method for the mimotopes Rp5‐L and Rp5‐C. Sample composition: (A) 0.30% (m/v) AESlyte HR 3–10; (B) 0.70% (m/v) AESlyte HR 3–10, 1.0% (m/v) AESlyte SH 6–9; (C) 0.70% (m/v) PL 3–10, 1.0% (m/v) PL 5–6, all with 0.35% (m/v) MC. (B) and (C) contained also p*I* markers (4.22 and 7.05) to demonstrate the change in the pH gradient.

The synthesized mimotopes were separated in a first attempt using 0.30% (m/v) of a high resolution (HR) CA from AES covering pH 3–10, that is, HR AESlyte 3–10. Although both peptides were separated (Figure 1A), a further improvement in the resolution was targeted to allow for a superior distinction not only between both peptides but also from possible impurities or interferences. Therefore, 0.70% (m/v) HR 3–10 was combined with a narrow pH range CA of AES, that is, super high resolution (SH) AESlyte covering pH 6–9. The selection of the narrow pH range AESlyte was governed by the in silico‐derived p*I*‐values of the mimotopes (see Table S1) and aimed to widen the pH gradient in the presumed focusing region of the peptides. However, the addition of 1.0% (m/v) SH 6–9 did not improve the electrophoretic resolution (R_S_). Moreover, two p*I* markers (p*I* 4.22 and p*I* 7.05) were added to demonstrate the spatial extension of this p*I* interval, which is an indicator of the slope of the pH gradient over this p*I* section. This p*I* interval covered 868 pixels and thus 42.4% of the total cartridge separation length of 2048 pixels. With the addition of SH 6–9, increased baseline noise within the focusing domain of the peptides was introduced (see Figure 1B).

To solve this limitation, a combination of PL CAs was tested in corresponding concentrations. The selected narrow pH range PL covered a considerably smaller pH region, that is, pH 5–6, compared to the SH AESlyte. This choice considered also the fact that acidic narrow pH range PLs actually seem to exceed their upper nominal pH limit (e.g., pH 6) markedly, whereas this effect is less pronounced for the lower nominal limit [63]. The addition of PL 5–6 is intended to improve the R_S_ of the mimotopes better than the SH AESlyte 6–9. When combining 0.70% (m/v) PL 3–10 with 1.0% (m/v) PL 5–6, the p*I* interval between 4.22 and 7.05 was indeed extended to 1198 pixels. The widening of the pH gradient exerted a pronounced effect on the focusing positions of the mimotopes. This is reflected by the considerably improved R_s_ of the peptides which grew by a factor of 3.0 (Figure 1C). Some baseline irregularities were observed between pixel positions 200 and 500, which is due to the CA composition. However, they do not interfere with the focusing position of the mimotopes.

#### 3.1.2 Optimized iCIEF Separation

**FIGURE 2:**
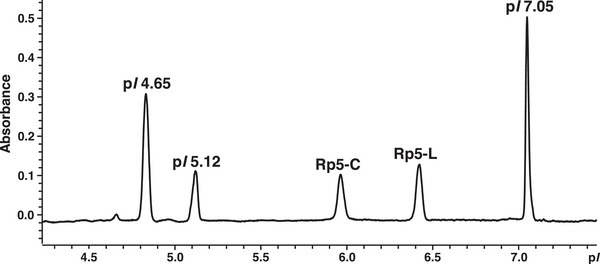
Imaged capillary isoelectric focusing (iCIEF) of Rp5‐L and Rp5‐C with p*I* markers. Sample composition: 0.50% (m/v) PL 3–10; 1.0% (m/v) PL 5–6; 0.35% (m/v) MC; 10.4 µmol/L Rp5‐L; 18.2 µmol/L Rp5‐C;150.7 µmol/L p*I* 4.65; 104.4 µmol/L p*I* 5.12 and 44.0 µmol/L p*I* 7.05. Focusing conditions: +0.5 kV for 1.0 min, +1.0 kV for 1.0 min, and +3.0 kV for 16.0 min. Separation was performed in a whole column imaging detection (WCID) cartridge with fluorocarbon coating (id 200 µm). The x‐axis was converted to p*I*‐scale. Since only two reference markers can be assigned in the software (i.e., 5.12 and 7.05), this causes a slight deviation of the focusing position of p*I* marker 4.65 from its nominal p*I*.

Finally, a combination of 0.50% (m/v) PL 3–10 and 1.0% (m/v) PL 5–6 was selected for further iCIEF measurements (Figure 2) since it maintained the R_S_ (see section 3.2.6) and reduced the electric current during the initial focusing steps (see section 2.5). In addition, three commercial p*I* markers were selected. The acidic markers flanked the mimotopes closer than the one used in Figure 1, thus allowing for an improved experimental determination of their p*I* [64, 65]. The applied CA combination provided baseline separation of both mimotopes and all p*I* markers (Figure 2). Apparent p*I* values were calculated from measurements done on five different days with independently prepared samples and five injection replicates on each day. Therefore, these p*I* values consider also the experimental variability between different days. Using the close flanking markers with p*I* 5.12 and p*I* 7.05, apparent p*I*s of 5.99 ± 0.01 and 6.47 ± 0.01 (mean ± 95% confidence interval (CI)) were calculated for Rp5‐C and Rp5‐L, respectively, by interpolation. This corresponded best with values predicted by the IPC p*I* calculator (Table S1). Evidently, the cyclization via the disulfide bond decreased the apparent p*I* of Rp5‐C by ∼0.5 units in comparison to Rp5‐L. Consistently, iCIEF allows to address structural differences between cognate oligopeptides.

### 3.2 Validation

The following sections address a single‐laboratory validation of the iCIEF method according to the ICH guideline Q2(R2) [58]. Validation considered specificity/selectivity, repeatability and intermediate precision of pixel position, peak area and peak height of the focused analytes, the working range and the linearity based on calibration data, the inter‐day comparability of calibration slopes, the limit of detection (LOD) and limit of quantification (LOQ) as well as a robustness testing and a suitability test. A determination of the trueness of the method was not considered since the tested mimotopes are not commercial and neither a certified reference material nor a primary method is currently available.

#### 3.2.1 Specificity/Selectivity

According to the ICH guideline Q2(R2) [58] impurities, matrix compounds but also related substances can be employed to demonstrate specificity. Isolated impurities or matrix compounds for spiking the synthesized mimotope products are not available. However, the iCIEF measurement of a blank has demonstrated the absence of (detectable) interferences (see Figure S4). Moreover, guideline Q2(R2) outlines, that “*For separation techniques …. specificity can be demonstrated by the resolution of two compounds which elute close to each other*”. This can be realized by analyzing “… *materials structurally similar to or closely related to the analyte*…” [58]. In the present case, the two mimotopes with their highly cognate primary structure represent such related compounds. Thus, the specificity of the iCIEF method was characterized by the R_s_ between Rp5‐C (29.0 µmol/L) and Rp5‐L (11.5 µmol/L). For these concentrations, the optimized iCIEF method provided a resolution of 6.96 ± 0.06 (mean ± 95% CI) for the mimotope pair (*n* = 24).

#### 3.2.2 Repeatability and Intermediate Precision

The repeatability of the iCIEF method was determined by six consecutive injection replicates per day applying a sample, which contained the optimized CA combination, Rp5‐L, Rp5‐C, the p*I* markers, and 0.35% (m/v) MC. Repeatability testing was done on four different days with independently prepared samples of identical composition (Table S2). The repeatability (given as coefficient of variation (CV) for six injection replicates per day) for peak heights and peak areas was <2.20% and <4.20%, respectively. The repeatability in terms of the pixel position was 1–15 pixels, corresponding to a CV of 0.04%–0.94% (Table S2). The intermediate precision of peak heights and peak areas was <6.75%. The intermediate precision of the pixel position of focused zones was 3–21 pixels, corresponding to 0.67%–1.20% (Table S3). This demonstrates that intermediate precision shows no substantial deterioration compared to repeatability. The intermediate precision of the experimentally determined p*I* for the mimotopes was <0.10% (CV).

#### 3.2.3 Calibration and Working Range

Calibration standard solutions included the optimized CA combination, Rp5‐L, Rp5‐C, the three p*I* markers, and 0.35% (m/v) MC. As the ICH guideline Q2(R2) required at least five calibration concentrations [58], a calibration with six different concentrations was done. Different concentration ranges, all in all between 0.70 and 150.72 µmol/L, were tested for the analytes (Table S4). Calibration levels for the different analytes seem diverse but were selected to achieve comparable peak areas. Calibration concentrations were evenly spaced across the tested working range. Calibration standards were prepared independently immediately prior to their injection, avoiding serial dilutions. Instead of repetitively injecting the individual calibration concentrations, standards were injected in the order of increasing concentration in three cycles, which obviates a misinterpretation of possible time‐dependent effects, such as calibration drifts, as non‐linearity [66]. This resulted in three replicates of either concentration (in total 18 data points). In total, two independent calibration series were prepared and measured on different days (day 1 and day 8). In CIEF, the linearity for peak areas is superior to peak heights as demonstrated before [67] which was also observed in the current case (data not shown). Calibration lines based on peak areas were derived by an ordinary least squares method (OLSM) [58] assuming a first‐order linear regression according to *y* = b_o_ + b_1_ · *x* (with b_o_ referring to the intercept and b_1_ addressing the slope), thereby avoiding its forcing through the origin [66]. OLSM requires several prerequisites to provide valid results, including (i) absence of outliers or influential points, (ii) normal distribution of residuals, (iii) homoscedastic residuals (i.e., errors), (iv) independence of observed errors, and (v) a linear relation between the predictor (*x*_i_) and the dependent variable (*y*_i_) [68, 69]. Although experimental deviations from this frame are frequent, most papers in analytical chemistry lose sight of (some of) these aspects when dealing with calibration data [69].

##### 3.2.3.1 Screening Calibration Data for Outliers

**FIGURE 3:**
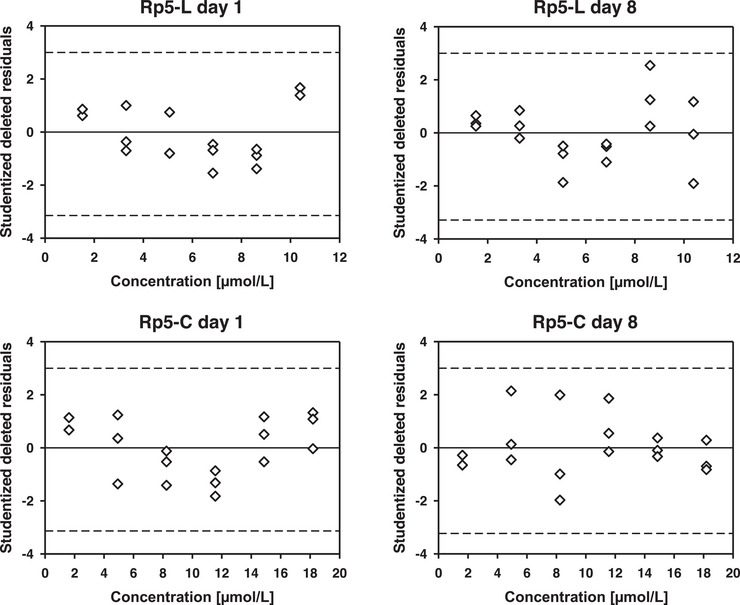
Studentized deleted residual (SDR) plots for Rp5‐C and Rp5‐L. SDRs are plotted against their respective calibration concentration to reveal possible outliers. For concentrations where apparently only two symbols are visible in the figure, differences between two data points and thus SDRs are too small to be depicted distinctly. Dotted reference lines at ‐3.0 and +3.0 indicate the critical values of the SDR for the detection of a y‐outlier (see section 3.2.3.1) and refer to a significance level of α = 0.01.

In regression, x‐ and y‐outliers can be distinguished. Testing for x‐outliers based on their leverage value h_ij_ [70] is of limited use in the presented calibration since concentrations were evenly distributed [71], whereas y‐outliers (i.e., large residuals) may act as influential points with impact on b_o_, b_1_, on the predicted y‐values $\left({\hat{y}}_{i}\right)$ and on SD [72]. Studentized deleted residual (SDR) plots were applied in addressing y‐outliers (Figure 3 and Figure S5). In addition, Cook's distance (Cook's D), standardized difference in fit (DFFITS), and standardized difference in fits of beta (DFBETAS) were applied, which simultaneously address x‐ and y‐outliers [66, 70]. Further information and values are given in Table S5. Indeed, for all calibration points, leverage values were <0.22 and thus below the critical value for the OLSM with n = 18 (data not shown). According to the literature, absolute values >3.0 for SDR (with α = 0.01; https://www.ibm.com/docs/en/cognos-analytics/12.0.0?topic=tests‐studentized‐residual‐test), >0.724 for Cook's D, and >1.0 for DFFITS and DFBETAS indicate an outlier [66, 70]. A calibration value was considered an outlier in case it exceeded all stated thresholds. Values for individual peptides and p*I* markers of both calibration series (days 1 and 8) are listed in Table S5. Based on the results for SDR (−5.329) (see Figure S5), Cook's D (1.108) and DFFITS (−2.452) as well as DFBETAS for the intercept (1.138) and for the slope (−2.025) (Table S5), the peak area for the highest calibration concentration of the second measurement cycle for p*I* 5.12 (day 8) was addressed as a y‐outlier (see section 3.2.3.2). This outlier occurred not due to a degradation of p*I* 5.12 since in the last measurement cycle of this day the signal for the highest calibration concentration of p*I* 5.12 resulted in no extreme value. Moreover, no comparable effect was observed in the first calibration series (day 1). After the elimination of this presumed outlier, only DFFITS (1.325) and DFBETAS for the slope (1.126) slightly exceeded their respective threshold (Table S5).

##### 3.2.3.2 Testing Residuals for Normal Distribution

**FIGURE 4:**
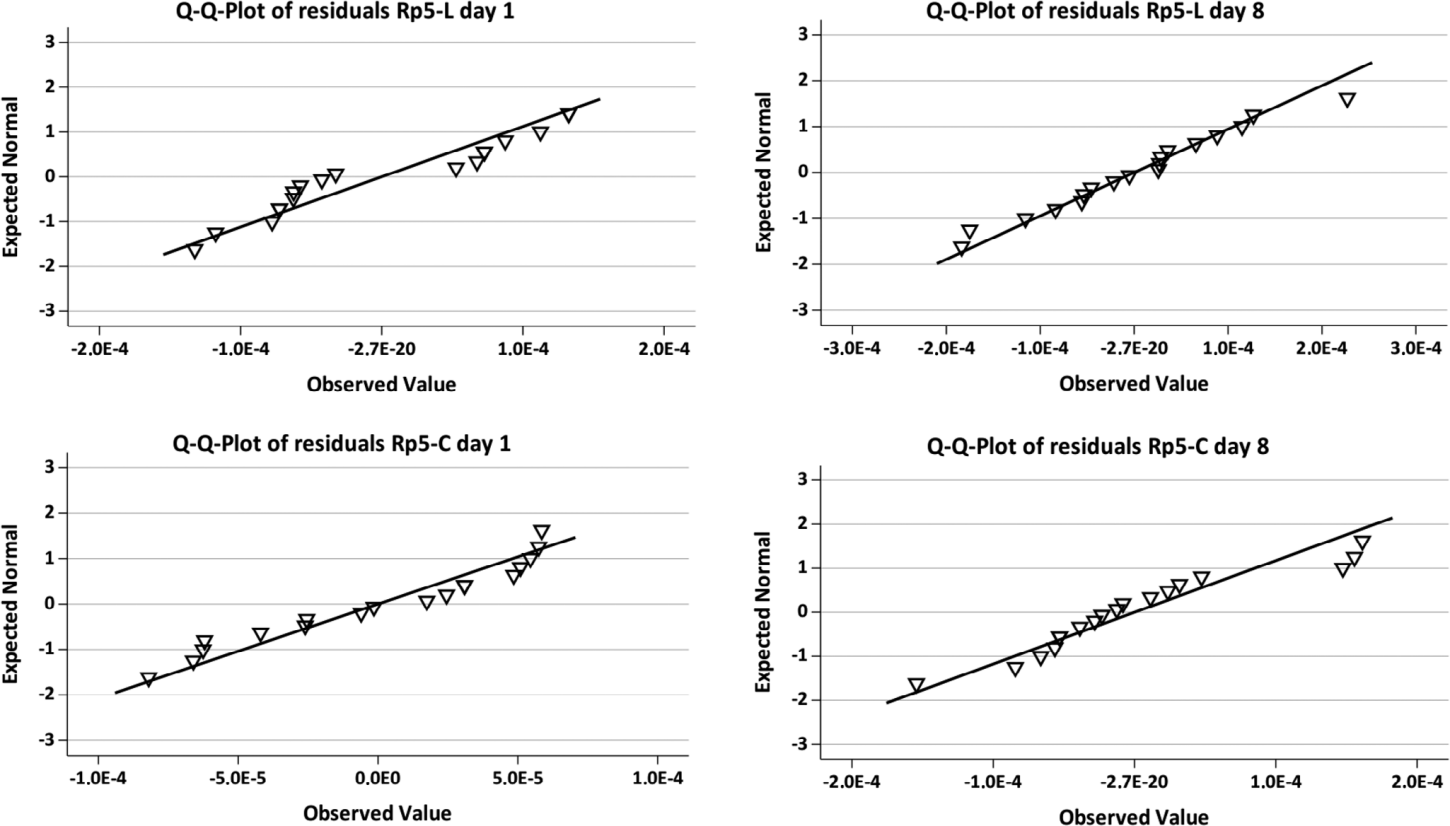
Normality testing for Rp5‐C and Rp5‐L by quantile‐quantile plots of residuals derived from the calibration where peak areas of the mimotopes were plotted against their respective concentrations. Tested concentrations refer to Table S4.

Normal distribution of calibration residuals was evaluated by (i) quantile‐quantile (Q‐Q) plots [66], by (ii) (bootstrapped) excess kurtosis and skewness of the distribution of the residuals, and by (iii) statistical tests, i.e., Lilliefors‐ and Shapiro‐Wilk (SW) test. Q‐Q plots for the peptides are shown in Figure 4, whereas Q‐Q plots for p*I* markers are given in Figure S6. Skewness and excess kurtosis of residuals are provided together with the respective standard error (SE) (Table S6). For p*I* 5.12 (day 8), both skewness (−1.397 ± 1.131; arithmetic mean ± 95% CI) and excess kurtosis (4.688 ± 2.190) were significantly different from zero, indicating a negatively skewed and leptokurtic distribution of residuals for linear first‐order regression. 95% CIs were calculated from SEs and t_0.05, DF = 17_, that is, 2.11. For a quadratic regression model, the high excess kurtosis was maintained (4.606 ± 2.190), whereas skewness approached zero (−0.300 ± 1.131) (Table S6). The elimination of the y‐outlier identified in section 3.2.3.1 resulted in a normal distribution of residuals for linear first‐order regression of p*I* 5.12 (day 8) with a skewness of 0.008 ± 1.166 and an excess kurtosis of −0.113 ± 2.254. For all other analytes, normality could not be rejected and was thus accepted (Table S6). However, since the SE of skewness and excess kurtosis depend only on the sample size n, this approach might lead to a wrong estimation of population parameters. Instead, bootstrapping using the bias‐corrected and accelerated (BCa) method has been recommended for calculating SEs and CIs [73]. Bootstrapping in SPSS using the BCa method with *n* = 1000 and α = 0.05 resulted in 95% CIs for skewness and excess kurtosis reflecting the population parameters more realistically [73], covering zero for all analytes and thus indicating normality of residuals. The outlier seems of less impact in the BCa approach. However, when the presumed outlier was included an increased bias and SE for BCa‐derived skewness and excess kurtosis were still encountered for the residuals of p*I* 5.12 (day 8). After the elimination of the outlier, BCa‐related bias, SE, and 95% CI for the residuals of p*I* 5.12 (day 8) were improved (Table S6A).

Finally, the normal distribution of residuals was evaluated with the Lilliefors‐ and the SW test. The SW test indicated a deviation from normality for p*I* 5.12 (day 8; *p *= 0.025) (Table S7), which is due to the identified y‐outlier (section 3.2.3.1) since its elimination resulted in a normal distribution of the residuals (*p *= 0.833) (Table S7). This explains why the BCa 95% CIs of skewness and excess kurtosis cover zero (Table S6A), since the described effect is indeed outlier‐related and not due to an inherent deviation from normal distribution. For Rp5‐C and Rp5‐L (both for day 1), *p*‐values close to α = 0.05, that is, *p *= 0.082 and *p *= 0.044, respectively, occurred for first‐order regression, whereas the Lilliefors test showed this only for Rp5‐L (*p *= 0.060) (Table S7). As shown later, both calibration series (Rp5‐C, Rp5‐L) failed also in the linearity testing with MFT over the entire concentration range for day 1 (section 3.2.3.5 and Table S13). For a quadratic regression approach, normality was confirmed for all analytes, except for p*I* 5.12 (day 8) when the outlier was included (Table S7).

##### 3.2.3.3 Testing for Homoscedasticity

An increase in the signal variances is frequently observed over extended concentration ranges in photometry, HPLC, and CE [74]. Testing for homogeneity of variances (= homoscedasticity) may comprise an evaluation via residual plots or by the Breusch‐Pagan test. Although regression coefficients (b_o,_ b_1_) estimated by OLSM will not be biased in the case of heteroscedasticity, their SEs may be affected and entail invalid CIs and statistical results [75, 76]. This will also affect the calculated uncertainty of the analyte concentration over the calibration range. In the case of heteroscedasticity, instead of data transformation or weighted least squares, heteroscedasticity consistent (HC) estimators for the SE of the regression coefficients, for example, HC3, can be used. Further details are outlined elsewhere [75, 76]. For Rp5‐L and Rp5‐C, the Breusch‐Pagan test proved homogeneity over the calibration range, with *p* > 0.05 in either case (Table S8). For the p*I* markers, homoscedasticity was confirmed for p*I* 4.65 and p*I* 7.05 (both day 8) and for p*I* 5.12 (day 8; after elimination of the outlier) (Table S9). The higher tendency toward heteroscedasticity of the p*I* markers might be related to their more extended concentration range (Table S4) and/or the fact that the p*I* markers were focused within a section of the pH gradient with presumably decreasing concentration of narrow pH range CA species. The latter might influence the signals of different calibration standards and injection replicates as well. After linearity testing with the MFT (section 3.2.3.5), homogeneity was re‐tested for the modified concentration ranges. Except for p*I* 5.12 (day 1), which was only slightly below the critical threshold (*p *= 0.044), homoscedasticity was then proven for all p*I* markers and series (Table S9).

##### 3.2.3.4 Test for Lack of Autocorrelation

Independence of residuals, that is, lack of autocorrelation, was tested against the chronology of injections with the Durban‐Watson (DW) test to exclude time‐dependent effects. As the individual calibration concentrations were not injected repetitively (section 3.2.3), possible time‐dependent effects, such as calibration drifts, can be addressed, which may remain unnoticed otherwise [66, 77]. A DW‐statistic close to 2.0 is considered to confirm a lack of autocorrelation. Based on the lower (d_L_) and upper (d_U_) critical DW‐value, that is, d_L_ = 1.158, d_U_ = 1.391 (for *n* = 18; *m* = 1 (due to the fact that only one regressor is considered); α = 0.05) (https://www3.nd.edu/~wevans1/econ30331/Durbin_Watson_tables.pdf) [66], the critical lower and upper bounds for the DW‐statistic were determined with 1.391 (=d_U_) and 2.609 (=4‐d_U_) [78]. Calculated DW‐statistics for Rp5‐L and Rp5‐C (days 1 and 8) were between 1.430 and 1.969, respectively, and thus within these bounds (Table S10). Consistently, autocorrelation and thus time‐dependent effects can be excluded for the mimotope residuals. The same holds for the p*I* markers (DW‐statistics: 1.760–2.534), except for p*I* 4.65 (day 8), since its DW‐statistic (1.164) is situated in the zone of statistical indifference between d_L_ and d_U_ leading to an inconclusive result [78]. Lack of autocorrelation was also confirmed for p*I* 5.12 (day 8) after the elimination of the outlier (DW‐statistic: 2.256), considering the changed critical DW‐bounds due to the lower sample size (*n *= 17) (Table S11).

##### 3.2.3.5 Test for Linearity

The regression coefficients b_o_ and b_1_, which are derived from a statistical sample of size n, are employed to predict the population parameters β_o_ (intercept) and β_1_ (slope). Dividing the regression coefficient by its respective SE provides the corresponding t‐statistic for either coefficient [78]. The alternative hypothesis H_1_ (intercept ≠ 0) is accepted if *p *< 0.05. For p*I* 4.65 and p*I* 5.12 (day 1, respectively) and p*I* 7.05 (for both days) the intercept b_o_ deviated only randomly from zero. For all other regression lines, b_o_ differs from zero (*p *< 0.05) (Table S12).

According to the ICH guideline Q2(R2), “*A linear relationship between the analyte concentration and the response should be evaluated across the range of the analytical procedure*…”. In addition to the regression function by OLSM, *r* ^2^, b_o_, and b_1_ should be provided [58]. However, the frequent use of *r* ^2^ in proving linearity is a misconception [66, 77, 79, 80] since *r* ^2^ addresses the correlation between the predictor (concentration) and the variable (signal), but not linearity [68]. Instead, linearity has to be tested by non‐statistical, that is, graphical, or by statistical approaches. MFT and LOF tests are adequate statistical tools for this purpose [68, 81]. In place of the simplified IUPAC version of the MFT, the genuine approach of Mandel was applied here for linearity testing for reasons outlined elsewhere [60, 81]. The LOF test requires a minimum of three replicates for each calibration level [60], as done in the present case. Both tests are based on the Fisher‐Snedecor F‐test principle [80, 81], but differ in the calculation of the numerator and the denominator [60]. Importantly, MFT addresses deviation from linearity by comparing a linear first‐order model only to an alternative quadratic regression model, whereas the LOF test follows a different concept [68]. The MFT and the LOF test were both performed with α = 0.01. In case the MFT becomes significant, the highest calibration concentration has to be deleted stepwise and the MFT is repeated with the remaining data set until the quadratic approach becomes insignificant. The strategy of stepwise elimination of the highest concentration and subsequent re‐calculation is also applicable for the LOF test in case of significant results [81].

Discrepancies in the linearity of both calibration series (days 1 and 8) may be related to the independent preparation of the calibration standards. For the mimotopes Rp5‐L and Rp5‐C, the MFT delivered slightly different results for days 1 and 8. To achieve linearity (*p* ≥ 0.01), the highest calibration concentration had to be eliminated for both Rp5‐L and Rp5‐C for day 1. In the case of the LOF test, this was not required for Rp5‐C (day 1). For day 8, linearity was confirmed over the entire calibration range of Rp5‐L and Rp5‐C by both the MFT and the LOF test (Table S13). For p*I* 4.65 and p*I* 5.12, the MFT proved linearity over the entire concentration range, except for p*I* 4.65 on day 8. With the LOF test, the highest concentration of these p*I* markers or in one case the three highest concentrations had to be eliminated to ensure linearity, except for p*I* 5.12 (day 8) (Table S13). The biggest discrepancy between both test procedures was observed for p*I* 7.05, where MFT proved linearity over the entire calibration range, while for the LOF test, the highest concentration had to be eliminated for day 8. For day 1, even after the elimination of the three highest calibration concentrations, the LOF test did not provide a linear response for p*I* 7.05 (Table S13).

*Conformity of results for the linearity tests*

For the mimotopes and the p*I* marker 5.12, conformity of the MFT and the LOF test results was achieved (except for Rp5‐C and p*I* 5.12, both on day 1). This was not the case for p*I* 4.65 and p*I* 7.05 (Table S13). However, as shown previously, narrow pH range CAs contain also species beyond their nominal pH domain, although at lower concentrations. Thus, PL 5–6 CA species most likely “leak” in regions of lower and higher pH (see section 3.1.1) [63]. We assume, that the mimotopes and p*I* 5.12 were focused within the pH domain of the gradient which is dominated by the narrow pH range CA species. The other p*I* markers may get focused either in the presumed transition region from narrow to wide pH range CAs (i.e., p*I* 4.65) or in a zone where only wide pH range CA species are expected (p*I* 7.05). Together with their higher calibration concentrations in comparison to the mimotopes, this could be of relevance for calibration results. In case, the signal increase with rising concentrations does not follow a parabolic course, the LOF test will presumably be more susceptible to this kind of non‐linearity than the MFT. Moreover, the LOF test is influenced by the method precision, this means that precise data make rejection of linearity with the LOF test more likely [68]. Altogether, this might explain the described deviations in some MFT and LOF test results.

##### 3.2.3.6 Comparison of Regression Slopes of Two Independent Calibration Series

**FIGURE 5:**
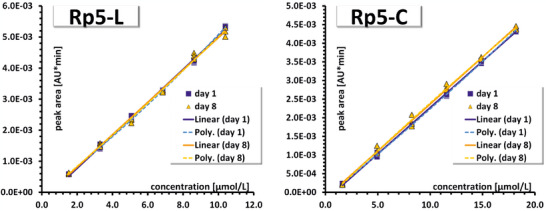
Comparison of calibration curves performed on day 1 and day 8 for Rp5‐L and Rp5‐C. Calibration standards on days 1 and 8 as well as the different concentrations were prepared independently and immediately prior to their injection. Further details are given in the text. Annotations in the legend: Linear, refers to a first‐order regression line according to *y *= b_0_ + b_1_·*x*; Poly., refers to a quadratic regression line according to *y *= b_0_ + b_1_·*x* + b_2_·*x* ^2^. The results of the statistical evaluation corresponding to this figure are provided in Tables S12 and S13.

The temporal stability of calibration results is scarcely addressed in method validations, although this provides valuable insights in (i) changes or drifts of the analytical equipment over time as well as in (ii) the inter‐day variability of the operator performance. Thus, it would be indicative of a reasonable frequency of (re‐)calibration of the instrument and may additionally serve as a quality tool for operator performance. An inter‐day comparison of calibration lines was done for the iCIEF method. Calibration standards were prepared independently for either calibration series (section 3.2.3). For each analyte, the calibration curves of day 1 and day 8 were pairwise compared first for their homogeneity of variances and then for the equality of their slopes. The statistical comparison of the slopes was first done by Welch's *t*‐test, which assumes different variances for the compared calibration curves. The DFs of Welch's *t*‐test were calculated according to the Aspin‐Welch approach [61]. In the homogeneity testing as well as in the calculation of the t‐statistic according to Welch's *t*‐test, variances (= SD^2^) are involved. In both statistical tests, the variance of the regression model (i.e., s^2^_y/x_) (see Table S12), but not the variance of the calibration slope has to be applied for reasons outlined elsewhere [61]. The slopes of both calibration series were additionally compared by the pooled *t*‐test which assumes homogeneity of residual variances. In case s^2^_y/x_ for calibrations of day 1 and day 8 differ only randomly, the pooled *t*‐test is more sensitive in revealing differences between the slopes. The decision about homoscedasticity of s^2^_y/x_ was made by the Fisher‐Snedecor F‐test at a significance level of 0.01 [61]. For Rp5‐L and p*I* 4.65, identical s^2^_y/x_ values for both calibration series were confirmed (*p *= 0.252 and *p *= 0.044, respectively). For Rp5‐C and p*I* 7.05, the calculated F‐statistic was only slightly higher than the critical value (*p *= 0.014 and *p *= 0.012, respectively). In the case of p*I* 5.12, variances were different irrespective of whether the outlier was included or eliminated, with *p *< 0.0003 in either case (Table S12). For Rp5‐L and Rp5‐C, Welch's *t*‐test resulted in equivalent slopes for both series (*p *= 0.369 and *p *= 0.386, respectively) (Figure 5 and Table S12). For p*I* 7.05, equivalent slopes were confirmed as well (*p *= 0.442), whereas, for p*I* 4.65 and p*I* 5.12, the slopes of the calibration series (days 1 and 8) were different with either *t*‐test (*p *< 0.001) (Figure S7 and Table S12). Elimination of the outlier in p*I* 5.12 (day 8) did not change the outcome (Table S12). The pooled *t*‐test provided *p*‐values equivalent to Welch's *t*‐test (differing only in the third or even lower digit). The consistency of the statistical results for (i) Welch's *t*‐test and (ii) the pooled *t*‐test (Table S12) is related to the fact that the statistical sample size n of both calibration series was identical [61].

Several parameters may contribute to differences in s^2^_y/x_ and in the slopes of both calibration series, including (i) the independent preparation of the calibration standards and (ii) possible changes in the performance of the instrument as well as of the WCID cartridge. In the case of (i), this might occur due to slight differences in the actual concentrations of mimotopes, of p*I* markers, and/or of the narrow and wide pH range CAs, and therefore influence the signal intensity and/or the UV background. Narrow pH range CA species will also extrude to pH domains outside their nominally specified pH range [63, 82]. Consistently, the observed effects can differ between the individual parts of the pH gradient, that is, in sections (i) containing only PL 3–10, (ii) containing both PL 3–10 and PL 5–6, and (iii) in the transition region where concentrations of the CA species of PL 5–6 start fading (see section 3.2.3.5). For the homogeneity of s^2^_y/x_, no general trend in terms of the respective focusing region (wide or narrow pH range CA region) was revealed (Table S12) and the encountered effects are most likely related to the independent preparation of the iCIEF calibration standards, which comprised delicate pipetting of small volumes. However, in terms of the equality of slopes for both calibration series, the results were more consistent. Only the acidic p*I* markers (4.65 and 5.12) were affected by differences in the slope between the tested series on days 1 and 8 (Table S12). We assume that this is due to their focusing position within the presumed transition region where PL 5–6 CA species start to decrease.

#### 3.2.4 LOD and LOQ

LOD and LOQ were determined for the mimotopes and the p*I* markers, according to the ICH guideline Q2(R2) using either (i) the S/N approach or (ii) the calculation via the slope of the related calibration curve for signal intensities, that is, peak heights [58]. Mostly disregarded, the applied concept of noise determination has to be specified since different approaches in noise evaluation will result in altered values for LOD and LOQ as stated elsewhere [83]. The signal fluctuation of noise represents a Gaussian‐shaped probability density function characterized by an arithmetic mean (set to zero) and its SD. Contrary to the approach where the entire noise range, that is, the difference between maximum and minimum noise signals, is taken, the core noise was considered in the present case, which cuts extreme noise values and approximates four times the SD of noise. This corresponds to a 95% CI of the noise signal. In accordance with the European Pharmacopoeia [84], only noise signals on the positive side of the zero‐noise value (i.e., of the arithmetic mean of noise) make up the baseline noise (i.e., 2×SD of noise). LOD and LOQ are calculated based on a S/N of 3:1 and 10:1, respectively [58]. The baseline noise was determined from blank runs. Baseline fluctuations were considered ±100 pixels around the average pixel position of the focused zone of either analyte, which was determined before. This spatial width of the selected individual baseline sections complies with the request of the European Pharmacopoeia, that is, considered baseline sections should cover at least five times the peak width measured in 50% of the peak height [84]. For the S/N approach, a sample containing Rp5‐L, Rp5‐C, and p*I* markers close to their respective LOQ was measured 10 times. LOD and LOQ were then derived from the determined noise, the peak heights, and the related analyte concentrations.

For the approach using the calibration slope, the SD is calculated from analyte signals close to the estimated LOQ. These SDs are then multiplied by 3.3 (for LOD) or by 10 (for LOQ) and divided by the slope, respectively [58]. The slope of calibration curves based on peak heights was determined on two different days (days 1 and 8) and subjected to the MFT. Concentration ranges conforming to linearity were used for slope calculation (data not shown). Based on the calibration slopes of both days, LODs and LOQs were first calculated for individual days and then averaged. LODs and LOQs calculated with the different strategies are surveyed in Table 1. The LOD and LOQ for Rp5‐C are ∼1.5–1.7 times higher than for Rp5‐L, although both peptides contain the same number of aromatic amino acid residues (Table S1). The difference is either due to the cyclic structure of Rp5‐C and/or a slightly different background absorption of CA species in the respective focusing region since the number of distinct CA species varies over the different pH sections [63]. Electropherograms close to the calculated LOD and LOQ are shown in Figure S8.

**TABLE 1:** Comparison of the limit of detection (LOD) and the limit of quantification (LOQ) for mimotopes and p*I* markers calculated with complementary approaches.

Analyte	LOD [µmol/L]	LOQ [µmol/L]
**p*I* 4.56**	0.28^a^/0.85^b^	0.92^a^/2.57^b^
**p*I* 5.12**	1.58/1.53	5.27/4.64
**Rp5‐C**	0.29/0.31	0.96/0.95
**Rp5‐L**	0.24/0.18	0.79/0.53
**p*I* 7.05**	0.11/0.13	0.36/0.40

^a^ Calculated with the S/N approach [58].

^b^ Calculated from the slope of the regression line for peak heights and the SD of the peak heights for analyte concentrations close to LOQ: LOD = $\left(\frac{3.3\cdot SD_{signal}}{slope}\right)$ and LOQ = $\left(\frac{10\cdot SD_{signal}}{slope}\right)$ [58].

#### 3.2.5 Suitability Test and Acceptance Criteria

For the validated iCIEF method, specific suitability performance characteristics with related acceptance criteria were established, to demonstrate appropriate method performance and verify that both “*the measurement system and the analytical operations*” meet the intended purpose and allow to reveal “*an unacceptable performance*.” [58]. Therefore, after the WCID cartridge has been installed in the instrument and filled with the sample, the system suitability test starts with the acquisition of a profile scan of the WCID cartridge which is initiated via the software and shows the measured intensity against the pixels along the WCID cartridge. Intensity values between 8,000 and 15,550 absorbance units ensure an appropriate performance over the separation length of the WCID cartridge. In the second step, a model mixture (Table S14) is run under optimized iCIEF separation conditions. Considered acceptance criteria comprise the R_S_ of Rp5‐C and Rp5‐L (R_s_ > 6.50), the pixel position of the apex of p*I* 4.65 (pixel 370–400) and of p*I* 7.05 (pixel 1880–2000), and the peak height of p*I* 7.05 (0.315‐0.365 absorbance). Based on the peak height of p*I* 7.05 acceptance criteria for peak height ratios were defined for the other compounds of the suitability mix (Table S15) to ensure appropriate performance.

#### 3.2.6 Robustness

The robustness of the iCIEF method was tested by a deliberate variation of the key factor in (i)CIEF separations, that is, the CA composition. Thus, selected combinations of PL 3–10 (0.30%‐0.70% (m/v)) and PL 5–6 (0.50% and 1.0% (m/v)) were tested for their effect on the focusing position and the resolution (R_s_) of the mimotope pair (see Figure S9). A variation between 0.50% and 0.70% (m/v) PL 3–10 provided nearly identical focusing positions for the mimotopes (see section 3.1.2; Figure S9A,C). In case 0.30% (m/v) PL 3–10 was applied, the pixel interval between the mimotopes was increased by 15% and both mimotopes moved more toward the ends of the separation cartridge (Figure S9B). However, the resolution was only slightly improved since the peak widths became 6–10% broader due to a flatter pH gradient caused by a higher relative contribution and thus a more prominent occupation of the available separation distance in the WCID cartridge by CA species of PL 5–6. In all these cases, a combination with 1.0% (m/v) PL 5–6 was used.

A combination of 0.70% (m/v) PL 3–10 and 0.50% (m/v) PL 5–6 shifted both mimotopes to lower pixel positions thereby reducing the pixel interval between their focusing positions by 24.6% and the R_S_ by 11.8% compared to the optimized conditions (Figure S9D). The R_S_ was less affected since the peak widths became smaller. This was due to a steeper pH gradient in this region since the CA species of PL 3–10 became more dominant in comparison to the tested combinations with 1.0% (m/v) PL 5–6. Results of the robustness testing showed only marginal effects within the tested PL 3–10 range (0.50%–1.0% (m/v)) when maintaining the PL 5–6 content, but a stronger influence of PL 5–6 within 0.50% and 1.0%. However, even a reduction to 0.50% (m/v) PL 5–6 still provided an excellent R_S_, which was more than 4‐fold higher than the baseline resolution, thus proving a robust performance of the iCIEF method.

## 4 Concluding Remarks

Peptides constitute emerging biopharmaceuticals progressively gaining relevance. Therein, mimotopes represent an innovative model class. For the first time, iCIEF was successfully employed in the separation of two cognate mimotopes of a therapeutically relevant epitope exposed on a surface protein expressed in B‐cell‐related tumors, that is CD‐20 antigen. Both mimotopes were synthesized in‐house. Imaged CIEF provided a fast baseline separation of both mimotopes by using a combination of wide and narrow pH range CAs which selectively widened the pH gradient within their focusing domain. The observed p*I* difference is due to the cyclic structure of one mimotope formed by an intra‐chain disulfide bond, whereas the second mimotope takes a linear structure. Validation of the optimized iCIEF method was done according to the ICH guideline Q2(R2) and data were additionally subjected to a comprehensive statistical evaluation.

Results support the feasibility of iCIEF in the distinction and quantification of peptide mimotopes allowing also for the resolution of different conformations in case they establish different p*I*‐values. Upcoming concepts to combat cancer but also other diseases of increasing socio‐economic impact, such as neurodegenerative and allergic disorders, progressively focus on preventive vaccination or use strategies that boost endogenously expressed antibodies in the active treatment of the disease. In both cases, mimotopes set promising biopharmaceuticals. As shown, iCIEF constitutes a fast and cost‐efficient analytical tool for the characterization and quantification of mimotopes with future perspectives in testing the batch‐to‐batch consistency of commercial products. Thus, iCIEF offers a promising complementary approach adding to the analytical portfolio currently in use for the analysis of peptides and mimotopes. Comprehensive statistical evaluation of data contributes an important aspect, allows for an improved understanding of results, and deserves a more prominent role in future validation strategies.

## Author Contributions

**Georg Bloderer**: investigation, formal analysis, visualization, and writing—review & editing. **Luigi Grassi**: investigation and resources. **Chiara Cabrele**: investigation, supervision, visualization, resources, writing—original draft, and writing—review & editing. **Hanno Stutz**: conceptualization, formal analysis, supervision, visualization, project administration, writing—original draft, and writing—review & editing.

## Conflicts of Interest

The authors declare no conflicts of interest.

## Supporting information



Supporting Information

## Data Availability

The data that support the findings of this study are available in the supplementary material of this article
