# Supplementary material for: An Imaged Capillary Isoelectric Focusing Separation of the Linear and Cyclic Variants of a Mimotope of the Cancer‐Related CD20 Antigen–Validation and Statistical Evaluation
Source: J Sep Sci. 2024 Dec 30;48(1):e70054. doi: 10.1002/jssc.70054 (PMC11685353; doi:10.1002/jssc.70054)
Supplement: Supplementary file 1 — Supporting Information [file JSSC-48-e70054-s001.pdf]

Supporting information

An iCIEF separation of the linear and cyclic variants of a mimotope of the cancer-related CD20 antigen - validation and statistical evaluation

Georg Bloderer[1], Luigi Grassi[1], Chiara Cabrele[1], Hanno Stutz[1 *]

[1] Department of Biosciences and Medical Biology, University of Salzburg, A-5020 Salzburg, Austria

Corresponding author:

Assoc. Prof. Dr. Hanno Stutz, Department of Biosciences and Medical Biology, University of Salzburg, Hellbrunner Str. 34, A-5020 Salzburg, Austria

E-mail: ernst-hanno.stutz@plus.ac.at

Fax: +43-(0)662-8044-5751

Table of contents

1. In-house synthesis of mimotope peptides

1. 1 Chemicals for synthesis of mimotopes

1.3 Characterization of peptides with RP-HPLC-UV and MALDI-TOF-MS

Table S1. Properties of Rp5-L and Rp5-C.

Figure S1. RP-HPLC-UV and MALDI-TOF-MS data

Figure S2. Chemical structures and MALDI-TOF-MS spectra of the linear precursor of Rp5-C and cyclic Rp5-C

Figure S3. RP-HPLC-UV and MALDI-TOF-MS of Rp5-C

2. Validation of iCIEF method

2.1 Specificity/Selectivity

Figure S4: Blank for 0.50% (m/v) PL 3-10, 1.0% (m/v) PL 5-6 with 0.35% (m/v) MC

2.2 Repeatability and intermediate precision

Table S2. Repeatability

Table S3. Intermediate precision

2.3 Calibration and tested working range

Table S4. Concentrations of Rp5-L, Rp5-C, and p*I* markers

2.4 Screening calibration points of p*I* markers for outliers

Figure S5. Studentized deleted residual (SDR) plots for p*I* markers

Table S5. Statistical identification of possible outliers

2.5 Testing residuals for normal distribution

Figure S6. Quantile-quantile plots of residuals

Table S6. Sample parameter estimates for skewness and excess kurtosis of residuals

Table S6A. Bootstrap parameter estimates for skewness and excess kurtosis of residuals

Table S7. Testing residuals for normal distribution with Lilliefors test and Shapiro-Wilk (SW) test.

2.6 Test for homoscedasticity

Table S8. Homogeneity of calibration data tested for Rp5-L and Rp5-C

Table S9. Homogeneity of calibration data tested for p*I* markers

2.7 Test for lack of autocorrelation

Table S10. Testing residuals of Rp5-L and Rp5-C for lack of autocorrelation

Table S11. Testing residuals of p*I* markers for lack of autocorrelation

2.8 Intercept, slope, homogeneity of residual standard deviation ($s^2_{y/x}$) and comparison of regression slopes

Table S12. Calibration data for Rp5-L, Rp5-C and p*I* markers

Figure S7. Comparison of calibration slopes for measurement series (day 1 and day 8)

2.9 Linearity testing of calibration curves

Table S13. Linearity testing for Rp5-L, Rp5-C and p*I* markers

2.10 LOD and LOQ

Figure S8. Electropherograms close to calculated LOD and LOQ

2.11 Suitability test and acceptance criteria

Table S14. Composition of suitability test mix

Table S15: Acceptable relative peak heights

2.12 Robustness testing

Figure S9. Robustness testing based on the CA composition

References

1. In-house synthesis of mimotope peptides

1. 1 Chemicals for synthesis of mimotopes

Reagents and solvents for the peptide synthesis and analysis were of peptide-synthesis quality and HPLC grade. Fluorenylmethyloxycarbonyl (Fmoc)-protected amino acids, H-Glu(O-*tert*-butyl)-2-chlorotrityl-resin, H-Cys(trityl)-2-chlorotrityl-resin, 2-(1H-benzotriazole-1-yl)-1,1,3,3-tetramethyluronium hexafluorophosphate (HBTU), N,N-diisopropylethylamine (DIPEA), piperidine, N,N-dimethylformamide (DMF), N-methyl-2-pyrrolidone (NMP), dichloromethane (DCM), diethyl ether, ACN, ammonium acetate and trifluoroacetic acid (TFA) were purchased from Merck-Millipore (Germany), Biosolve (Valkenswaard, The Netherlands) and Iris Biotech GmbH (Marktredwitz, Germany). Triisopropylsilane (TIS), 1,2-ethanedithiol (EDT), thioanisole (TIA), and N-hydroxybenzotriazole (HOBt) were purchased from Sigma-Aldrich (Germany). α-Cyano-4-hydroxycinnamic acid was purchased from Acros Organics (Germany).

1.2 Synthesis of mimotopes

The peptides were assembled on an automatic peptide synthesizer Syro I from Biotage (Uppsala, Sweden) by using preloaded 2-chlorotrityl-resin and fluorenylmethoxycarbonyl (Fmoc) protecting group chemistry. The side-chain protecting groups were *tert*-butyl (for Asp, Glu, Thr), *tert*-butyl-oxycarbonyl (for Lys, Trp), and trityl (for Cys, Gln). The Fmoc deprotection was carried out with 25% (v/v) piperidine in DMF/NMP (70:30, v/v) for 3 min, and 12.5% (v/v) piperidine in DMF/NMP (70:30, v/v) for 12 min. The double couplings were accomplished with the mixture Fmoc-(amino acid)-OH/HOBt/HBTU/DIPEA (4:4:4:8 equivalents with respect to the resin linker) for 40 min. The peptides were cleaved from the resin with the mixture TFA/H_2O/TIA/EDT/TIS (90:3:1:3:3, v/v) for 3 h, precipitated from ice-cold diethyl ether, recovered by centrifugation (2700 x g, 4 °C, 6 min), and washed at least three times with neat ice-cold diethyl ether. The intramolecular disulfide bond in Rp5-C was obtained by air oxidation of the linear peptide in ammonium acetate (0.10 mol/L, pH 7.0) at a concentration of 0.80 mmol/L, followed by acidification with TFA and lyophilization.

1.3 Characterization of peptides with RP-HPLC-UV and MALDI-TOF-MS

The analytical HPLC equipment (model Ultimate 3000) and the analytical column (Syncronis C18, 100 Å, 5 µm, 250 mm x 4.6 mm) was both from Thermo Fisher Scientific (Germany). The UV detection was done at 220 nm. The mobile phase consisted of a binary system A/B, with A being 0.06% (v/v) TFA in water, and B being 0.05% (v/v) TFA in ACN. The flow rate was 1.5 mL/min. Peptide samples were prepared in water containing 0.10% (v/v) TFA. The analytical HPLC gradient used for Rp5-L was 5% B for 5 min, 5-55% B over 40 min. The analytical HPLC gradient applied for Rp5-C was 3% B for 8 min, 3-60% B over 35 min. MALDI-TOF-MS analysis of the synthetic peptides was performed in positive or negative mode on an Autoflex mass spectrometer from Bruker Daltonics (Germany) using α-cyano-4-hydroxycinnamic acid as matrix. Different ionization modes were used due to the analyte specific differences in ionization efficiency: positive ionization was most suitable for Rp5-L, whereas negative ionization was most suitable for Rp5-C.

Table S1. Properties of Rp5-L and Rp5-C.

Peptide	Primary sequence	Molecular mass [a] [Da]	Relative purity [b]	p*I* theor [c]	Molar absorption coefficient 280 nm [M^{-1} cm^{-1}] [g]
Rp5-L	*H*-QDKLTQ**WP**K**WLE**-*OH*	1571.80	89%	6.07[d]<br>6.25[e]<br>6.56[f]	11,380
Rp5-C	*cyclo-[C1,C15]*<br>*H*-CQDKLTQ**WP**K**WLE**GC-*OH*	1835.13 (linear)<br><br>1833.11<br>(cyclic; with disulfide bond considered)	87%	6.05[d]<br>6.25[e]<br>5.88[f]	11,500<br>(with disulfide bond considered)

The contained Trp residues allow for a peptide detection at 280 nm in iCIEF. The mimotope consensus motif is indicated bold and underlined. For Rp5-C, a Cys residue was added on the N-terminus, whereas the C-terminus was elongated with Gly-Cys. Gly was inserted to separate the mimotope consensus motif from the Cys involved in the disulfide bond. Residues added in Rp5-C to obtain a cyclic conformation are marked in red.

[a] isotope averaged mass calculated from primary sequence with Expasy (https://web.expasy.org/compute_pi/)

[b] relative purity calculated from %peak area by means of in-house RP-HPLC-UV results (see supplement section 1.3, Figures S1A and S3A).

[c] theoretical p*I* (p*I* theor) calculated by means of different algorithms, i.e.,

[d] Expasy Compute p*I*/Mw (https://web.expasy.org/compute_pi/),

[e] Protcalc (https://protcalc.sourceforge.net/),

[f] IPC isoelectric point calculator (http://isoelectric.org/calculate.php); this program averages the calculated p*I* of several algorithms specified on the website [1].

The disparity in the p*I* values predicted by the algorithms is caused by different pK_a values assumed for the amino acids by the respective calculation algorithms. However, the influence of the protein/peptide conformation and the effect of neighbor amino acids on the pK_a and p*I* of individual amino acids is not considered [2].

[g] molar absorption coefficient at 280 nm calculated for the primary sequence by means of https://protcalc.sourceforge.net/

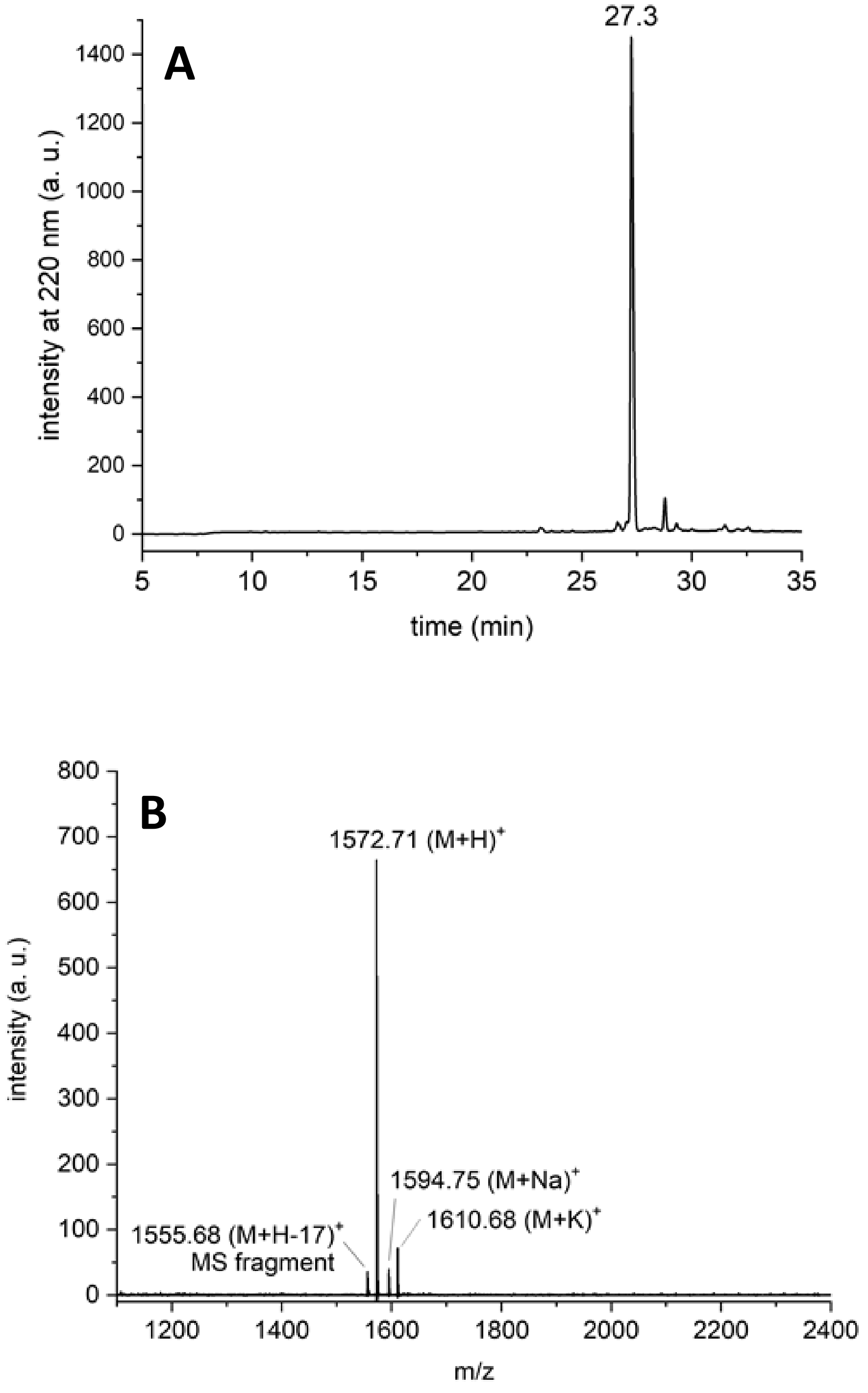

Figure S1. (A) RP-HPLC-UV and (B) MALDI-TOF-MS of Rp5-L (QDKLTQWPKWLE). (A) Rp5-L: t_R 27.3 min (purity: 89%). (B) 1572.71 refers to $(M+H)^+$ since measurement was done in positive ionization mode. M_{theor}/M_{found}: $[M+H]^+$ for $[C_{74}H_{111}N_{18}O_{20}]^+$ 1572.81/1572.71. $[M+Na]^+$ for $[C_{74}H_{110}N_{18}NaO_{20}]^+$ 1594.79/1594.75. $[M+K]^+$ for $[C_{74}H_{110}N_{18}KO_{20}]^+$ 1610.90/1610.68.

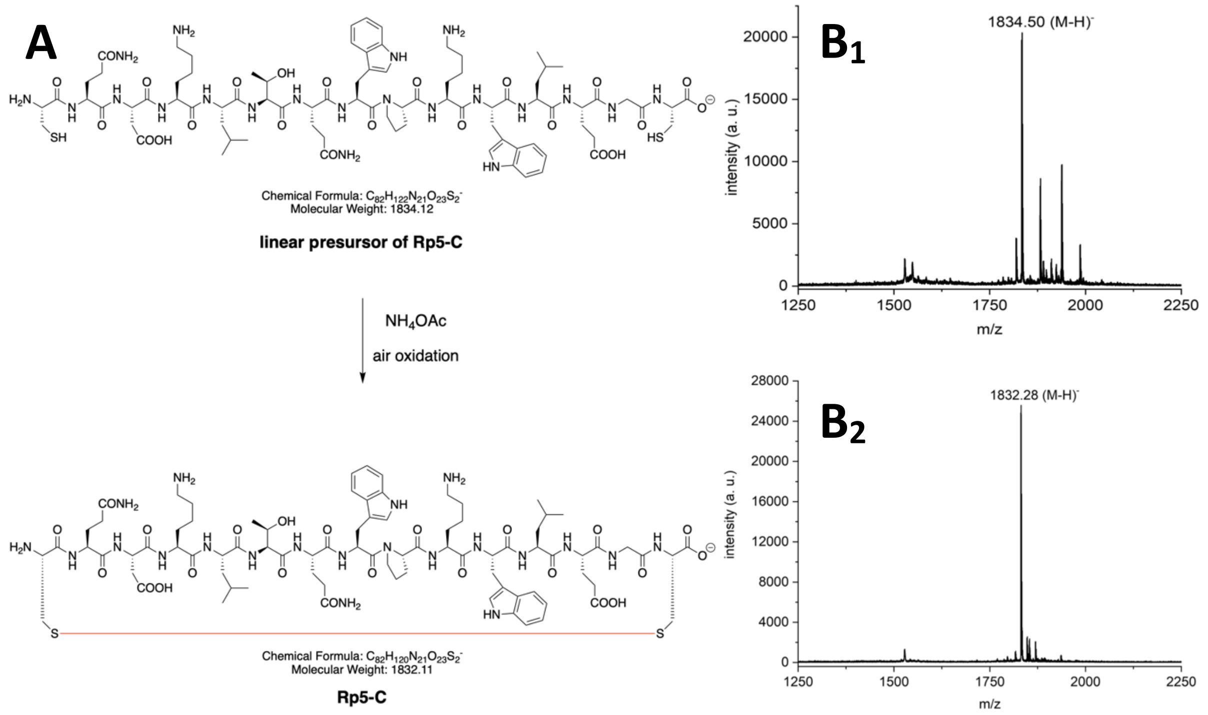

Figure S2. (A) Chemical structures of the linear precursor of Rp5-C and cyclic Rp5-C, and (B) their respective MALDI-TOF-MS spectra (in negative mode). Cyclization is introduced by disulfide bond formation (indicated in red). (B_1) M_{theor}/M_{found} before formation of disulfide bond 1834.12/1834.50. (B_2) M_{theor}/M_{found} after disulfide bond formation by air oxidation: $[M\text{-}H]^-$ for $[C_{82}H_{120}N_{21}O_{23}S_2]^-$ 1832.11/1832.28.

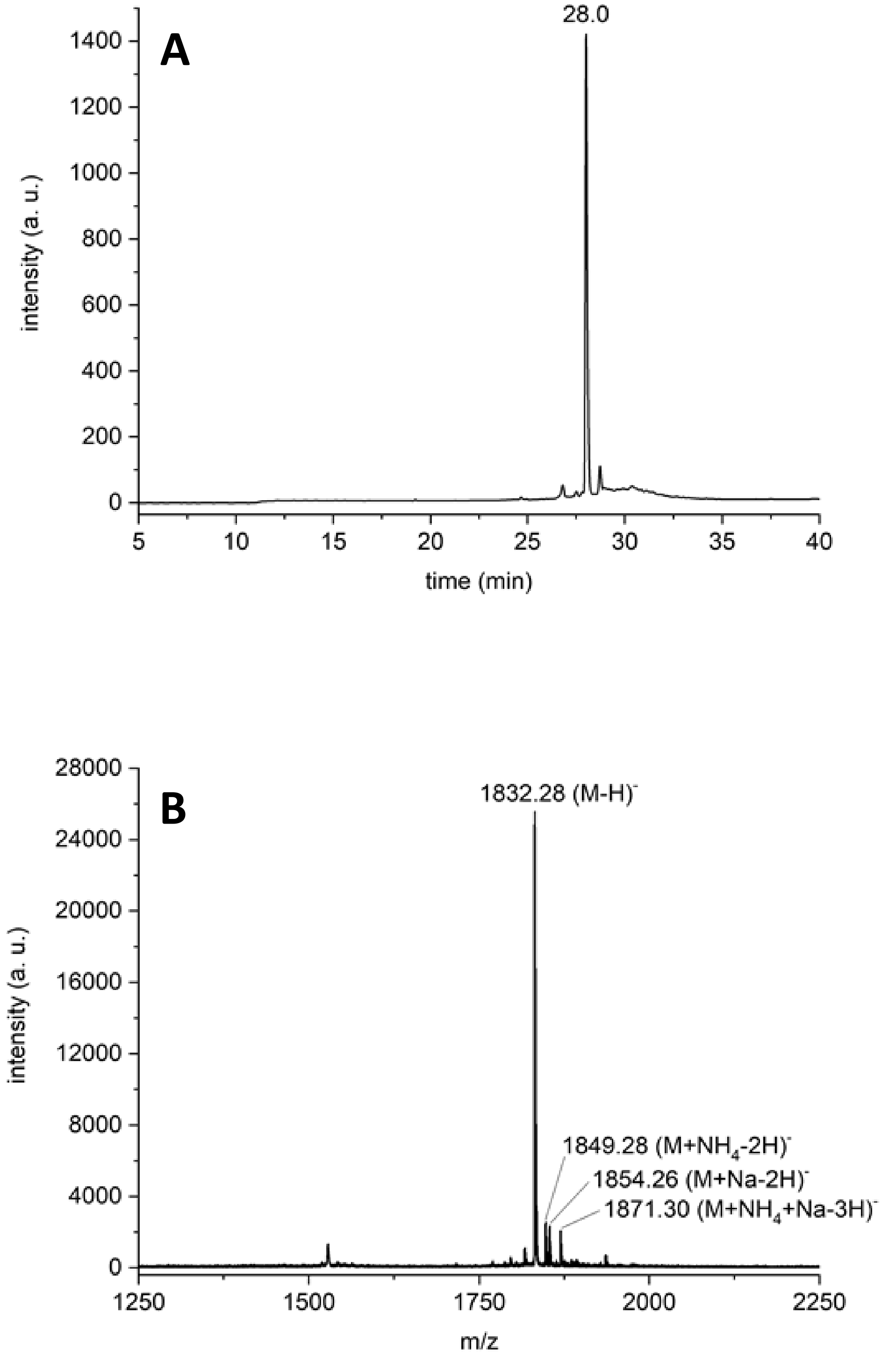

Figure S3. (A) RP-HPLC-UV and (B) MALDI-TOF-MS of Rp5-C (CQDKLTQWPKWLEGC). (A) Rp5-C: t_R 28.0 min (purity: 87%). (B) 1832.28 refers to $(M - H)^-$ since measurement was done in negative ionization mode. M_{theor}/M_{found}: $[M\text{-}H]^-$ for $[C_{82}H_{120}N_{21}O_{23}S_2]^-$ 1832.11/1832.28. $[M+NH_4\text{-}2H]^-$ for $[C_{82}H_{123}N_{22}O_{23}S_2]^-$ 1849.14/1849.28. $[M+Na\text{-}2H]^-$ for $[C_{82}H_{119}N_{21}NaO_{23}S_2]^-$ 1854.09/1854.26. $[M+NH_4+Na\text{-}3H]^-$ for $[C_{82}H_{122}N_{22}NaO_{23}S_2]^-$ 1871.12/1871.30.

2. Validation of iCIEF method

2.1 Specificity/Selectivity

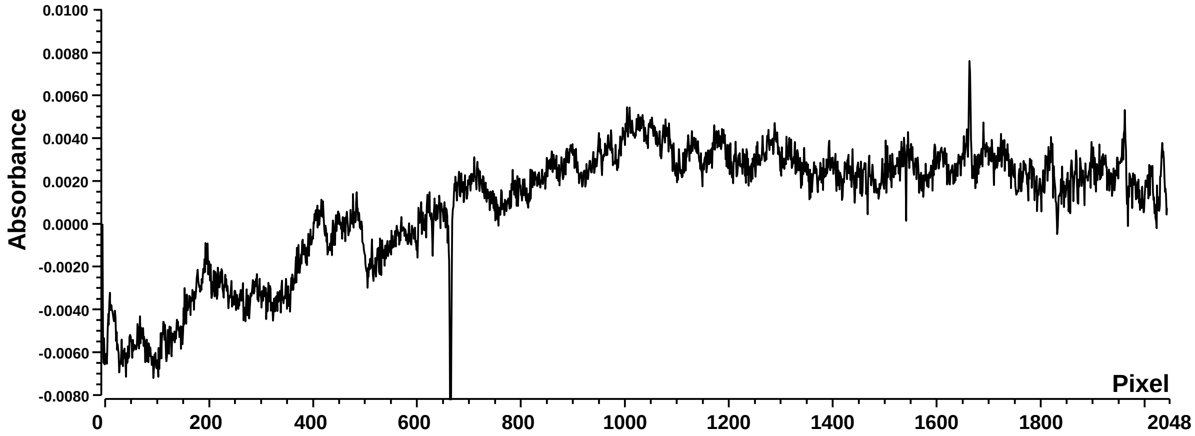

Figure S4: Blank for 0.50% (m/v) PL 3-10, 1.0% (m/v) PL 5-6 with 0.35% (m/v) MC without p*I* markers. Electropherogram of a blank proves the absence of signals which interfere with Rp5-C and Rp5-L. Baseline was acquired over the entire length of the WCID cartridge.

2.2 Repeatability and intermediate precision

Table S2. Repeatability determined from six consecutive injections of the same sample given as coefficient of variation (CV) of peak area, peak height, and pixel position after focusing. Measurements were done on four different days with independently prepared samples of corresponding composition. Values provide the range of the repeatability observed for four measurement series.

Analyte	Peak area CV (%) (n=6, per day)	Peak height CV (%) (n=6, per day)	Pixel count (focused position) CV (%) (n=6, per day)
**p*I* 4.65**	0.72 – 2.90	0.53 – 1.75	0.14 – 0.91
**p*I* 5.12**	0.34 – 1.79	0.75 – 1.46	0.19 – 0.89
**Rp5-C**	1.55 – 4.18	0.46 – 2.14	0.10 – 0.94
**Rp5-L**	1.04 – 3.74	1.22 – 2.17	0.07 – 0.92
**p*I* 7.05**	0.62 – 2.75	0.56 – 1.14	0.04 – 0.76

Table S3. Intermediate precision determined on four different days with six injections, respectively, given as coefficient of variation (CV) of peak area, peak height and pixel position after focusing.

Analyte	Peak area CV (%) (4 days) [a)]	Peak height CV (%) (4 days) [a)]	Pixel count (focused position) CV (%) (4 days) [a)]
**p*I* 4.65**	6.23	5.92	0.67
**p*I* 5.12**	2.71	3.52	0.71
**Rp5-C**	4.45	3.26	1.20
**Rp5-L**	6.44	6.71	1.04
**p*I* 7.05**	4.77	3.06	1.09

[a)] Samples of corresponding concentration were prepared independently on the individual days.

2.3 Calibration and tested working range

Table S4. Concentrations of Rp5-L, Rp5-C, and p*I* markers in the six independently prepared standard solutions employed in the calibration thus defining the tested working range. This working range of the iCIEF method was subjected to linearity testing subsequently.

Calibration standard	p*I* 4.65 [µmol/L]	p*I* 5.12 [µmol/L]	Rp5-L [µmol/L]	Rp5-C [µmol/L]	p*I* 7.05 [µmol/L]
**1**	3.92	12.98	1.52	1.62	0.70
**2**	33.28	31.26	3.30	4.93	9.36
**3**	62.64	49.54	5.07	8.24	18.02
**4**	92.00	67.82	6.84	11.56	26.68
**5**	121.36	86.10	8.62	14.87	35.34
**6**	150.72	104.43	10.39	18.19	44.00

2.4 Screening calibration points of p*I* markers for outliers by means of studentized deleted residuals (SDRs)

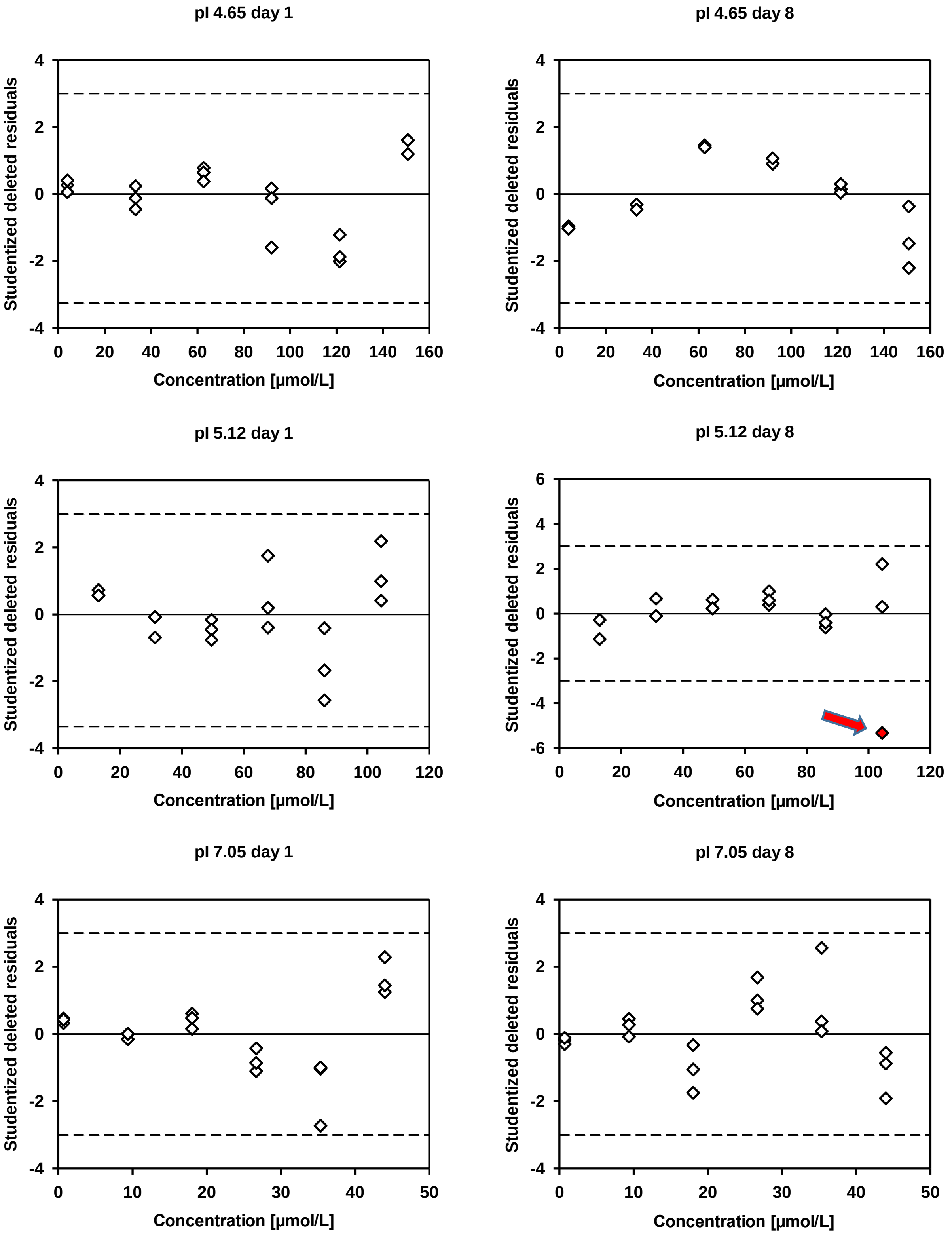

Figure S5. Studentized deleted residual (SDR) plots for p*I* markers. SDRs are plotted against their respective calibration concentration and are employed to reveal possible outliers. For concentrations where apparently only two symbols are visible in the figure, differences between two data points and thus SDRs are too small to be depicted distinctly. Dotted reference lines at -3.0 and +3.0 refer to the critical values of the SDR for the detection of a y-outlier (see section 3.2.3.1). The stated SDR thresholds refer to a significance level α=0.01 (see https://www.ibm.com/docs/en/cognos-

analytics/12.0.0?topic=tests-studentized-residual-test). For p*I* 5.12 (day 8) the highest concentration in the second measurement cycle is indicated as a (possible) y-outlier (symbol marked in red and additionally indicated by a red arrow). The calculation of the SDRs is explained in **Table S5**.

Table S5. Statistical identification of possible outliers within the calibration series (day 1 and day 8) by means of Cook´s distance, SDR (see also **Figures 3 and S5**), DFFITS and DFBETAS. Values of the respective parameters refer to calibration data with peak area representing the dependent variable. For the facility of inspection, only the highest absolute value (either as minimum (min.) or maximum (max.)) of the calibration series is given, respectively. In case, one of the related thresholds is violated (see section 3.2.3.1 in the manuscript), the corresponding influential data point is specified in the footnote of this table additionally. A calibration value has to exceed all stated thresholds to become considered as an outlier.

Analyte	Day	Cook´s D (max. value)	SDR (max. or min. value)	DFFITS (max. or min. value)	DFBETAS for intercept (max. or min. value)	DFBETAS for slope (max. or min. value)
Rp5-L	1	0.266	1.673	0.769	0.394	0.635
	8	0.331	2.542	-0.879	0.422	0.725
Rp5-C	1	0.178	-1.824	0.611	0.522	-0.505
	8	0.205	2.142	0.708	0.669	-0.467
p*I* 4.65	1	0.249	-2.017	0.740	-0.285	0.611
	8	0.415	-2.205	-1.014 [b]	-0.477	-0.838
p*I* 5.12	1	0.409	-2.568	1.006 [c]	-0.467	0.831
	8	1.108	-5.329	-2.452	1.138	-2.025
	8 w/o [a]	0.641	2.556	1.325 [d]	-0.702	1.126 [d]
p*I* 7.05	1	0.436	-2.732	1.049 [e]	-0.396	0.867
	8	0.333	2.560	-0.881	0.338	-0.728

[a] Calculation without (w/o) the outlier that was assigned in **Figure S5**. The outlier refers to the highest concentration (104.43 µmol/L) of the 2nd measurement cycle.
[b] 150.72 µmol/L, last injection; [c] 104.43 µmol/L, last injection; [d] 104.43 µmol/L, 1st injection; [e] 44.00 µmol/L, last injection

Cook´s D — Cook´s distance. Considers the residual and the leverage h_{ij} of each data point when testing whether individual calibration points are influential (see section 3.2.3.1).

SDR — Studentized deleted residual (= externally studentized- or Jackknife residual). After deletion of the ith calibration point, a new regression line is calculated. The residual of this data point i from the new regression line where it was not included is determined. This residual is then divided by its standard error. The SDR is a measure for the effect the elimination of a data point i has on its own prediction. Large residuals are indicative of outliers/influential points.

DFFITS — Standardized difference in fits. Measure for the difference in the predicted response ($\hat{y}_i$) if the ith data point, i.e., an individual calibration value, is (i) included in and (ii) excluded from the calculation. DFFITS values are z-transformed. $\hat{y}_i$ refers to the peak area calculated with the regression equation for the respective concentration.

DFBETAS Standardized difference in fits of beta. Difference for estimated regression coefficients $\hat{\beta}_o$ (intercept) and $\hat{\beta}_1$ (slope) if the ith data point, i.e., an individual calibration value, is (i) included in or (ii) excluded from the calculation. DFBETAS were calculated for all individual calibration values. DFBETAS values are z-transformed.

2.5 Testing residuals of p*I* markers for normal distribution by quantile-quantile (Q-Q) plots, skewness and excess kurtosis and by Lilliefors- and Shapiro-Wilk test

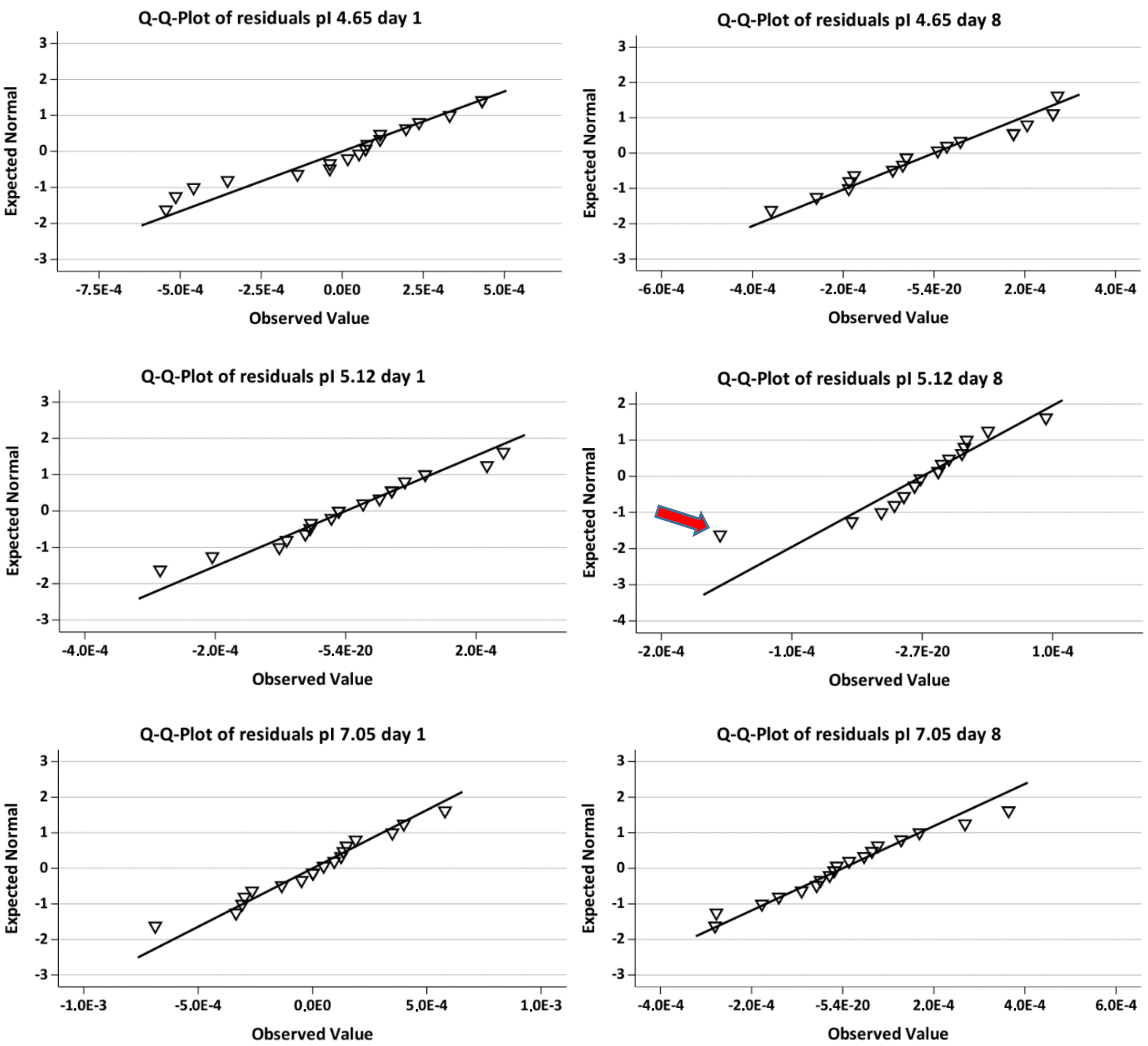

Figure S6. Quantile-quantile plots of residuals derived from the calibration where peak areas of p*I* markers were plotted against their respective concentrations. Tested concentrations refer to **Table S4**. For p*I* 5.12 (day 8) the highest concentration in the second measurement cycle is indicated as a (possible) y-outlier (symbol indicated by a red arrow)**.** This is consistent with the results for SDRs (see **Figure S5**) and with the relevant statistical parameters given in **Tables S5** and **S6**.

Table S6. Sample parameter estimates for skewness and excess kurtosis of residuals. For skewness and excess kurtosis, a value close to zero points to normal distribution of data [a)]. Normality testing for residuals of mimotopes and of p*I* markers based on (i) a first order linear regression model ($y = b_o + b_1 \cdot x$) and (ii) a second order regression model ($y = b_o + b_1 \cdot x + b_2 \cdot x^2$). For p*I* 5.12 (day 8) the skewness and excess kurtosis are calculated both with and without (w/o) the assumed outlier (see section 3.2.3.1, **Figures S5** and **S6**, and **Table S5**). This will examine whether the outlier has an influence on the data distribution (characterized via skewness and excess kurtosis).

Analyte	Day	Skewness				Excess Kurtosis			
		Linear 1st order		2nd order quadratic		Linear 1st order		2nd order quadratic	
		Skewness	SE [c)]	Skewness	SE [c)]	Excess Kurtosis	SE [c)]	Excess Kurtosis	SE [c)]
**p*I* 4.65**	1	-0.528	0.536	-0.541	0.536	-0.493	1.038	-0.765	1.038
	8	-0.069	0.536	-0.142	0.536	-1.029	1.038	-0.446	1.038
**p*I* 5.12**	1	-0.176	0.536	0.008	0.536	0.455	1.038	2.299	1.038
	8	-1.397	0.536	-0.300	0.536	4.688	1.038	4.606	1.038
	8 w/o [b)]	0.008	0.550	0.261	0.550	-0.113	1.063	0.808	1.063
**Rp5-C**	1	-0.328	0.536	0.317	0.536	-1.364	1.038	-0.460	1.038
	8	0.663	0.536	0.038	0.536	0.237	1.038	0.831	1.038
**Rp5-L**	1	0.185	0.536	0.786	0.536	-1.552	1.038	0.850	1.038
	8	0.124	0.536	0.180	0.536	0.086	1.038	1.306	1.038
**p*I* 7.05**	1	-0.287	0.536	-0.656	0.536	0.408	1.038	1.130	1.038
	8	0.312	0.536	-0.143	0.536	0.266	1.038	1.064	1.038

[a)] contrary to kurtosis, where a value close to 3 is indicative of normal distribution.

[b)] w/o: without outlier (see **Figure S5** and **Table S5**).

[c)] The standard error (SE) of skewness and excess kurtosis depends only on the sample size n, respectively. Thus, SE is identical throughout the individual analytes for skewness and kurtosis, respectively, except for p*I* 5.12 day 8 without (w/o) the outlier.

Table S6A. Bootstrap parameter estimates for skewness and excess kurtosis of residuals with Bias-corrected and accelerated (= BCa) method [a)] based on 1000 samples. Normality testing for residuals of peptides and p*I* markers based on a first order linear regression model ($y = b_o + b_1 \cdot x$).

Analyte	Day	Skewness				Excess Kurtosis			
		Skewness of the sample	Bias [b)] BCa	SE [c)] BCa	95% CI BCa	Excess Kurtosis of the sample	Bias [a)] BCa	SE [b)] BCa	95% CI BCa
**p*I* 4.65**	1	-0.528	-0.013	0.396	-1.322-0.243	-0.493	0.283	1.164	-1.790-4.027
	8	-0.069	0.033	0.398	-0.880-0.761	-1.029	0.126	0.603	-1.838-1.461
**p*I* 5.12**	1	-0.176	0.082	0.516	-1.230-1.296	0.455	-0.206	0.929	-0.713-1.305
	8	-1.397	0.568	1.087	-3.107-2.275	4.688	-1.966	2.587	-1.591-2.926
	8 w/o [d)]	0.008	-0.051	0.440	-0.803-0.692	-0.113	0.062	0.947	-1.293-2.138
**Rp5-C**	1	-0.328	0.001	0.425	-1.177-0.473	-1.364	0.239	0.660	-1.997-2.799
	8	0.663	0.002	0.520	-0.077-1.547	0.237	0.259	1.554	-1.755-5.854
**Rp5-L**	1	0.185	0.009	0.485	-0.719-1.214	-1.552	0.260	0.686	-2.021-3.079
	8	0.124	-0.063	0.463	-0.577-0.675	0.086	-0.082	0.911	-1.281-1.865
**p*I* 7.05**	1	-0.287	0.065	0.528	-1.333-0.952	0.408	-0.249	1.022	-0.788-1.145
	8	0.312	-0.088	0.467	-0.578-0.965	0.266	-0.106	0.986	-0.944-1.713

a) The BCa method considers skewness and bias in the bootstrap distribution. For mathematical details see [3].
b) Bias between the parameter of the sample and bootstrap (BCa) estimates for skewness and excess kurtosis, respectively.
c) BCa standard error (SE) of skewness and excess kurtosis for bootstrap results.
d) Parameters calculated without the previously addressed outlier (see **Figure S5** and **Table S5**).

Table S7. Testing for normal distribution of residuals with Lilliefors test and Shapiro-Wilk (SW) test. Normality testing for residuals of mimotopes and of p*I* markers based on (i) first order linear regression model ($y = b_o + b_1 \cdot x$) and (ii) second order regression model ($y = b_o + b_1 \cdot x + b_2 \cdot x^2$).

Analyte	Day	1st order regression Lilliefors test *p*-value	1st order regression SW test *p*-value	2nd order regression Lilliefors test *p*-value	2nd order regression SW test *p*-value
**p*I* 4.65**	1	0.170	0.174	0.200	0.229
	8	0.200	0.360	0.200	0.616
**p*I* 5.12**	1	0.200	0.901	0.057	0.076
	8	0.169	0.025	0.091	0.014
	8 w/o [a)]	0.200	0.833	0.200	0.635
**Rp5-C**	1	0.200	0.082	0.200	0.714
	8	0.200	0.111	0.141	0.241
**Rp5-L**	1	0.060	0.044	0.200	0.441
	8	0.200	0.968	0.200	0.684
**p*I* 7.05**	1	0.200	0.910	0.200	0.385
	8	0.200	0.871	0.200	0.410

[a)] w/o p-values calculated without the presumed outlier.

2.6 Testing for homoscedasticity

Table S8. Homogeneity of calibration data tested for Rp5-L and Rp5-C with Breusch-Pagan test (α=0.05). Results comprise homogeneity (i) for the entire tested concentration range, and (ii) for the corrected concentration range considering the results of the MFT (with α=0.01) (see **Table S13**).

Peptide	Day	Entire range [a] [µmol/L]	Breusch-Pagan (homogeneity) [b]	Corrected range (MFT) [c] [µmol/L]	Breusch-Pagan (homogeneity) [d]
**Rp5-L**	1	1.52-10.39	*p*=0.169 passed	1.52-8.62	p=0.797 passed
	8		*p*=0.114 passed	/	/
**Rp5-C**	1	1.62-18.19	*p*=0.933 passed	1.62-14.87<br>1.62-11.56	p=0.338 passed<br>p=0.677 passed
	8		*p*=0.482 passed	/	/

[a] comprises the entire tested calibration range (see **Table S4**).
[b] results of Breusch-Pagan test for entire tested calibration range.
[c] comprises the calibration range with proven linearity according to the MFT (α=0.01) (**Table S13**).
[d] results of Breusch-Pagan test for the calibration range with proven linearity according to the MFT (α=0.01).

Table S9. Homogeneity of calibration data tested for p*I* markers with Breusch-Pagan test (α=0.05). Results comprise homogeneity (i) for the entire tested concentration range, (ii) for the corrected concentration range considering the results of the MFT (with α=0.01), and (iii) for p*I* 5.12 (day 8) after the elimination of the outlier (see **Table S13**).

**p*I* Marker**	**Day**	**Entire range [a] [µmol/L]**	**Breusch-Pagan (homogeneity) [b]**	**Corrected range (MFT) [c] [µmol/L]**	**Breusch-Pagan (homogeneity) [d]**
**4.65**	1	3.92-150.72	*p*=0.012 failed	/	3.92-121.36 [e] *p*=0.381 passed
	8		*p*=0.625 passed	3.92-92.0	*p*=0.176 passed
**5.12**	1	12.98-104.43	*p*=0.043 failed (bdl.) [g]	12.98-86.10 [f] 12.98-67.82 [e]	*p*=0.044 failed (bdl.) [g] *p*=0.123 passed
	8		*p*=0.003 failed	/	/
	8 w/o		*p*=0.081 passed [h]		
**7.05**	1	0.70-44.00	*p*=0.006 failed	/	0.70-35.34 [e] *p*=0.174 passed
	8		*p*=0.100 passed	/	/

[a] comprises the entire tested calibration range (see **Table S4**).
[b] results of Breusch-Pagan test for entire tested calibration range.
[c] comprises the calibration range with proven linearity according to the MFT (α=0.01) (**Table S13**).
[d] results of Breusch-Pagan test for calibration range with proven linearity according to the MFT (α=0.01) or for a calibration range that had to be reduced to ensure homogeneity of residuals.
[e] calibration range was reduced to ensure homogeneity of residuals.
[f] this concentration range corresponds with the confirmed linearity of the LOF test (see **Table 13**).
[g] although strictly speaking classified as “failed”, this may be considered as a statistical borderline (bdl.) situation due to the closeness of *p* to the significance level (0.05).
[h] calculated without (=w/o) the previously identified outlier

2.7 Test for lack of autocorrelation

Table S10. Test for lack of autocorrelation of residuals for Rp5-L and Rp5-C with Durban-Watson (DW) test (α=0.05).

Peptide	Day	Entire range [a] [µmol/L]	DW-statistic	Critical bounds [b] for DW-statistic	Zones of indifference [c]
**Rp5-L**	1	1.52-10.39	1.736 passed	lower bound: 1.391	lower region: 1.158-1.391
	8		1.766 passed		
**Rp5-C**	1	1.62-18.19	1.430 passed	upper bound: 2.609	upper region: 2.609 – 2.842
	8		1.969 passed		

[a] comprises the entire tested calibration range (see **Table S4**).
[b] critical bounds provide the upper and lower threshold of the Durban-Watson (DW) statistic (for the experimental setting with n=18). Values of the DW-statistic within these bounds lead to rejection of autocorrelation.
[c] Zones of indifference: in case the calculated DW-statistic is situated within the zone of indifference, the null hypothesis can neither be accepted nor rejected.

Table S11. Test for lack of autocorrelation of residuals for p*I* markers with Durban-Watson (DW) test (α=0.05).

p*I* Marker	Day	Entire range [a] [µmol/L]	DW-statistic	Critical bounds [b] for DW-statistic	Zones of indifference [c]
**4.65**	1	3.92-150.72	2.423 passed	lower bound: 1.391<br>w/o 1.381 [d]	lower region:<br>1.158-1.391<br>1.133-1.381 [f]
	8		1.164 indifferent		
**5.12**	1	12.98-104.43	2.534 passed		
	8		1.760 passed		
	8 w/o [e]		2.256 passed	upper bound: 2.609<br>w/o 2.619 [d]	upper region:<br>2.609 – 2.842<br>2.619-2.867 [f]
**7.05**	1	0.70-44.00	2.008 passed		
	8		2.306 passed		

[a] comprises the entire tested calibration range (see **Table S4**).
[b] Critical bounds provide the upper and lower threshold of the Durban-Watson (DW) statistic (for the experimental setting with n=18). Values of the DW-statistic within these bounds lead to a rejection of autocorrelation.
[c] Zones of indifference: in case the calculated DW-statistic is situated within the zone of indifference, the null hypothesis can neither be accepted nor rejected.
[d] refers to critical bounds without the outlier (n=17).
[e] DW-statistic calculated without (=w/o) the previously identified outlier (see **Figure S5** and **Table S5**). As evident from the results, neither an inclusion nor an elimination of the outlier causes autocorrelation.
[f] zones of indifference for the data set p*I* 5.12 (day 8) without the outlier (n=17)

2.8 Intercept, slope, homogeneity of residual standard deviation ($s^2_{y/x}$) and comparison of regression slopes

Table S12. Calibration data for Rp5-L, Rp5-C and p*I* markers over the tested concentration range, respectively, assuming a first order regression according to $y = b_o + b_1 \cdot x$ (OLSM).

Analyte	Tested concentration range [µmol/L]	Calibration day	Intercept b_o	Hypothesis H_o intercept $b_o = 0$ (α=0.05)	Slope b_1	Homogeneity of $s^2_{y/x}$ a) (α = 0.01)	Slope comparison with Welch´s t-test (α = 0.05; 2-sided)	Slope comparison with pooled t-test (α = 0.05; 2-sided)	r^2
**p*I* 4.65**	3.92-150.72	1	-8.91E-05	*p*=0.516 passed	7.95E-05	*p*=0.044 passed	*p*=4.82E-04 failed	*p*=4.64E-04 failed	0.995
		8	2.18E-04	*p*=0.022 failed	7.28E-05				0.997
**p*I* 5.12**	12.98.0-104.43	1	-6.99E-05	*p*=0.319 passed	4.38E-05	*p*=2.43E-04 failed<br>w/o *p*=5.80E-07 failed [b)]	*p*=1.79E-04 failed<br>w/o *p*=4.62E-04 failed [b)]	*p*=9.47E-05 failed<br>w/o *p*=3.76E-04 failed [b)]	0.991
		8	5.86E-05	*p*=0.042 failed	3.89E-05				0.998
		8 w/o [b)]	4.03E-05	*p*=0.027 failed	3.94E-05				0.999
**Rp5-C**	1.62-18.19	1	-2.14E-04	*p*=1.04E-07 failed	2.49E-04	*p*=0.014 passed	*p*=0.386 passed	*p*=0.385 passed	0.998
		8	-1.56E-04	*p*=0.002 failed	2.52E-04				0.997
**Rp5-L**	1.52-10.39	1	-2.90E-04	*p*=1.64E-05 failed	5.31E-04	*p*=0.252 passed	*p*=0.369 passed	*p*=0.370 passed	0.997
		8	-2.28E-04	*p*=9.93E-04 failed	5.21E-04				0.996
**p*I* 7.05**	0.70-44.00	1	-1.38E-04	*p*=0.318 passed	2.47E-04	*p*=0.012 passed	*p*=0.442 passed	*p*=0.441 passed	0.994
		8	1.86E-05	*p*=0.805 passed	2.43E-04				0.998

a) Homogeneity comparison of $s^2_{y/x}$ for the calibration data of day 1 and day 8 was done by calculation of an F-statistic according to $F = \frac{s^2_{y/x\,1}}{s^2_{y/x\,2}}$, whereby $s^2_{y/x\,1} > s^2_{y/x\,2}$. $s^2_{y/x\,1}$, $s^2_{y/x\,2}$ refer to the variance of residuals of the calibration curves measured on the different days.

b) 8 w/o refers to data of p*I* 5.12 (day 8) where the presumed outlier was eliminated.

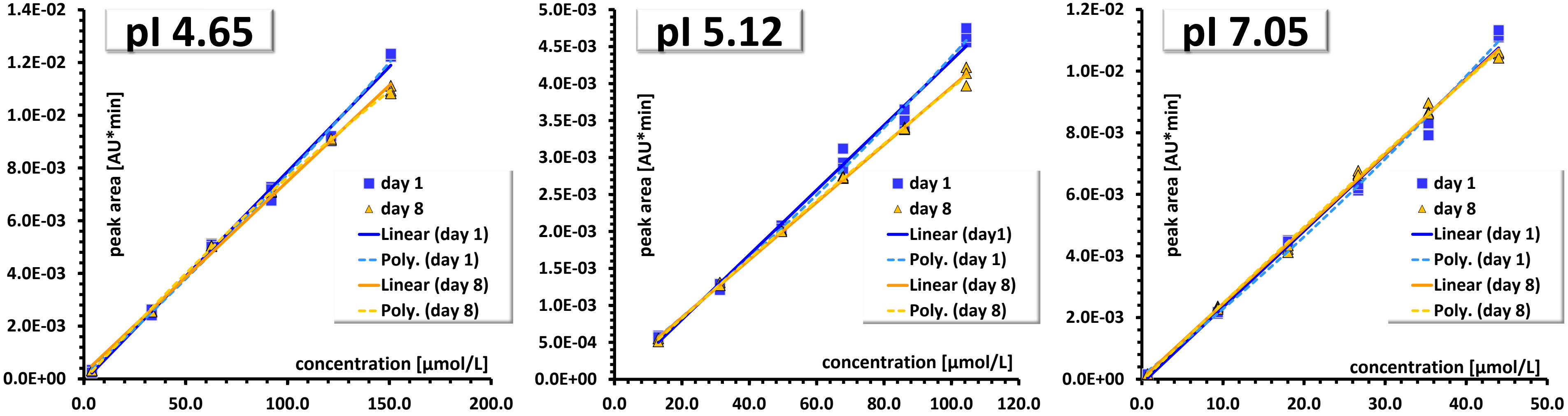

Figure S7. Comparison of calibration slopes for measurement series for p*I* markers performed on day 1 and day 8. Calibration standards of day 1 and day 8 as well as the different concentration levels were prepared independently immediately prior to their injection. Results of the statistical evaluation corresponding to this figure are provided in **Table S12**. Further details are given in the text. Inserted legends: Linear, refers to a first order regression line according to $y = b_0 + b_1 \cdot x$. Poly., refers to a quadratic regression line according to $y = b_0 + b_1 \cdot x + b_2 \cdot x^2$.

2.9 Linearity testing of calibration curves

Table S13. Linearity testing for Rp5-L, Rp5-C and p*I* markers with MFT and LOF test over the calibration range (with α=0.01, respectively) and comparison of the conformity of linearity results calculated with MFT and LOF test.

Analyte	Tested concentration range [µmol/L]	Calibration day	Linearity (MFT) [a] for entire concentration range	Linearity (MFT) [b] for reduced concentration range	Linearity (LOF) [c] for entire concentration range	Linearity (LOF) [d] for reduced concentration range	Conformity of MFT and LOF
**p*I* 4.65**	3.92-150.72	1	*p*=0.099 passed	/	*p*=4.10E-05 failed	3.92-121.36 µmol/L: *p*=0.0190 passed	no (LOF 1 conc. less)
		8	*p*=5.50E-07 failed	3.92-92.00 µmol/L: *p*=0.0766 passed	*p*=9.38E-07 failed	3.92-62.64 µmol/L: *p*=7.96E-06 failed	no (LOF >2 conc. less)
**p*I* 5.12**	12.98.0-104.43	1	*p*=0.069 passed	/	*p*=4.81E-03 failed	12.98-86.10 µmol/L: *p*=0.0519 passed	no (LOF 1 conc. less)
		8	*p*=0.144 passed	/	*p*=0.597 passed	/	yes
		8 w/o [e]	*p*=0.549 passed		*p*=0.037 passed		yes
**Rp5-C**	1.62-18.19	1	*p*=0.0024 failed	1.62 – 14.87 µmol/L: *p*=0.00947 failed (bdl.) [f] 1.62-11.56 µmol/L: *p*=0.916 passed	*p*=0.0186 passed	/	no (MFT 1-2 conc. less) [f]
		8	*p*=0.243 passed	/	*p*=0.499 passed	/	yes
**Rp5-L**	1.52-10.39	1	*p*=4.88E-04 failed	1.52-8.62 µmol/L: *p*=0.432 passed	*p*=1.08E-03 failed	1.52-8.62 µmol/L: *p*=0.817 passed	yes
		8	*p*=0.263 passed	/	*p*=0.0278 passed		yes
**p*I* 7.05**	0.70-44.00	1	*p*=0.0198 passed	/	*p*=7.38E-06 failed	0.70-18.02 µmol/L: *p*=5.60E-03 failed [g]	no (LOF >3 conc. less)
		8	*p*=0.176 passed	/	*p*=2.07E-03 failed	0.70-35.34 µmol/L: *p*=0.0211 passed	no (LOF 1 conc. less)

Abbreviations: bdl., borderline; conc., concentration; LOF test, lack-of-fit test; MFT, Mandel´s fitting test.

[a] evaluation of linearity with MFT considering the entire tested calibration range (see **Table S4**).
[b] concentration range with confirmed linearity (with MFT) after elimination of highest concentration(s) until $p \geq 0.01$.

[c] evaluation of linearity with LOF test considering the entire tested calibration range (see **Table S4**).
[d] concentration range with confirmed linearity (with LOF test) after elimination of highest concentration(s) until $p \geq 0.01$.
[e] calculation without (w/o) the outlier of p*I* 5.12 (day 8).
[f] although strictly speaking classified as “failed”, this represents a borderline (bdl.) situation due to the closeness of *p* to the significance level (0.01). Thus, a range between 1.62 and 14.87 µmol/L might be acceptable. Therefore, the conformity of MFT and LOF test is given as “MFT 1-2 conc. less”.
[g] LOF test failed in proving linearity although the highest three calibration concentrations were not considered.

2.10 LOD and LOQ

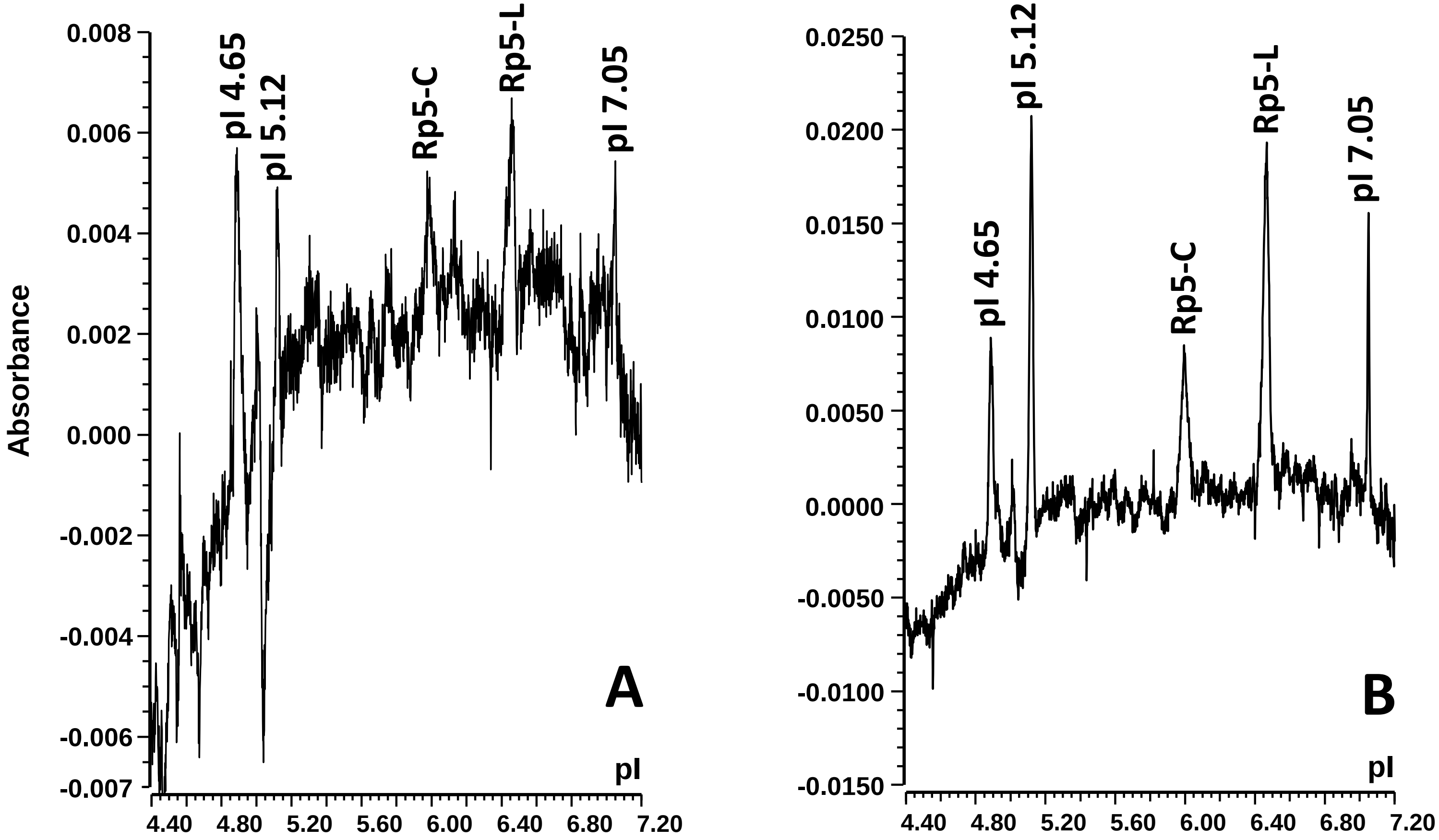

Figure S8. Electropherograms with mimotopes and p*I* marker concentrations close to their calculated LOD and LOQ. (A) Electropherogram close to LOD. Sample composition: 0.50% (m/v) PL 3-10; 1.0% (w/w) PL 5-6; 0.35% (m/v) methylcellulose; 0.57 µmol/L Rp5-L; 0.62 µmol/L Rp5-C; 1.31 µmol/L p*I* 4.65; 4.33 µmol/L p*I* 5.12; 0.23 µmol/L p*I* 7.05. (B) Electropherogram close to LOQ. Sample composition: 0.50% (m/v) PL 3-10; 1.0% (m/v) PL 5-6; 0.35% (m/v) methylcellulose; 1.52 µmol/L Rp5-L; 1.62 µmol/L Rp5-C; 3.92 µmol/l p*I* 4.65; 12.98 µmol/L p*I* 5.12; 0.70 µmol/L p*I* 7.05.

2.11 Suitability test and acceptance criteria

Table S14. Composition of suitability test mix with 0.50% (m/v) PL 3-10, 1.0% (m/v) PL 5-6 and 0.35% (m/v) MC.

Analyte	Concentration [μmol/L]
**p*I* 4.56**	62.5
**p*I* 5.12**	165.5
**Rp5-C**	16.9
**Rp5-L**	33.8
**p*I* 7.05**	22.0

Table S15: Acceptable relative peak heights (in %) of analytes related to signal height of p*I* 7.05 in the test mix.

Analyte	Relative peak height [%] [a)]	95% CI of relative peak height
**p*I* 4.56**	0.49	±0.05
**p*I* 5.12**	0.45	±0.04
**Rp5-C**	0.50	±0.03
**Rp5-L**	0.41	±0.05

[a)] Relative peak heights (%) are related to the peak height of p*I* 7.05 which was selected as reference signal since it gave the sharpest peak.

2.12 Robustness testing

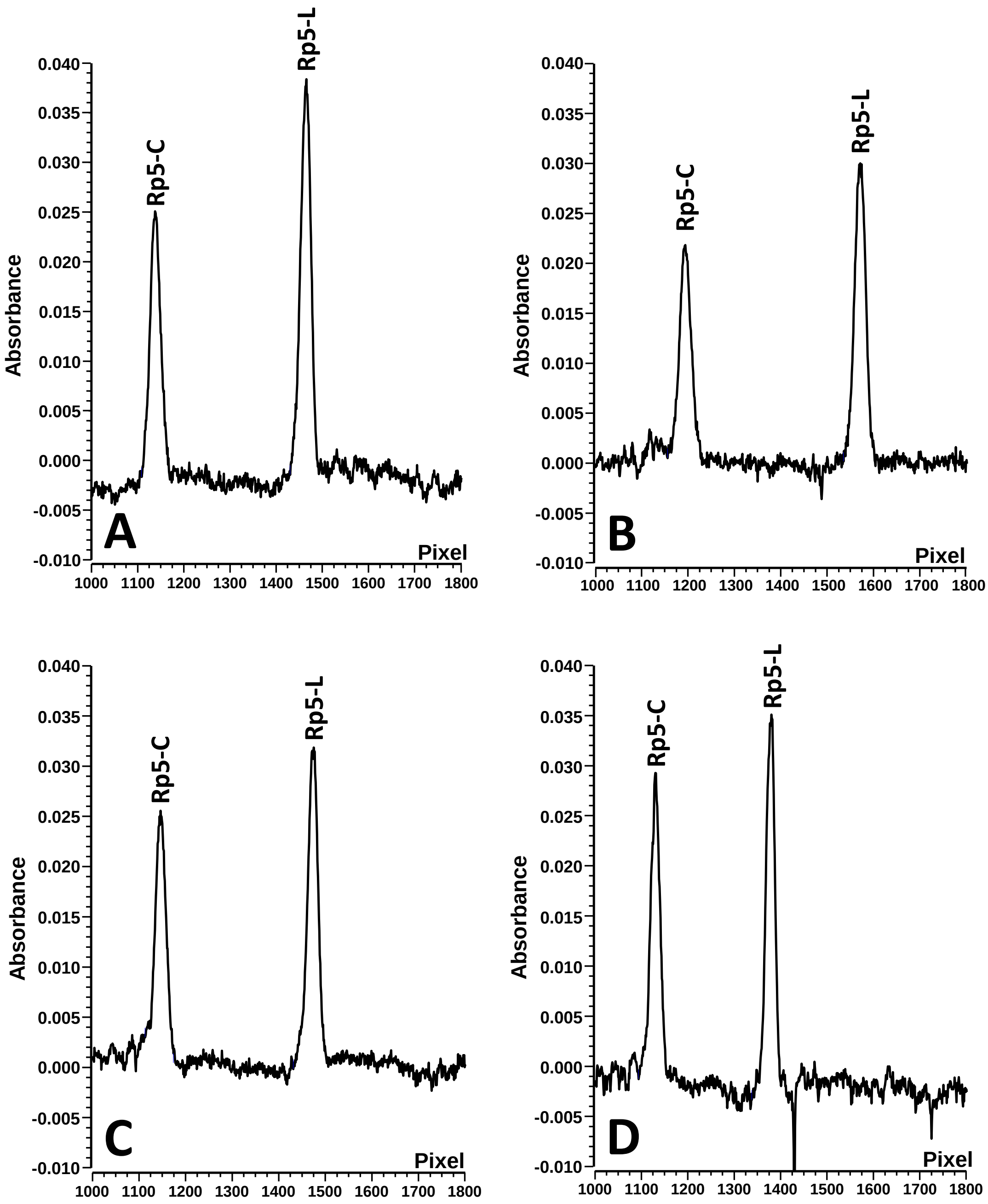

Figure S9. Robustness testing based on different CA compositions. (A) 0.50% (m/v) PL 3-10, 1.0% (m/v) PL 5-6, 0.35% (m/v) MC, i.e., optimized CA combination. (B) 0.30% (m/v) PL 3-10, 1.0% (m/v) PL 5-6, 0.35% (m/v) MC. (C) 0.70% (m/v) PL 3-10, 1.0% (m/v) PL 5-6, 0.35% (m/v) MC. (D) 0.70% (m/v) PL 3-10, 0.50% (m/v) PL 5-6, 0.35% (m/v) MC. All samples were prepared independently immediately prior to their use.

References

[1] Kozlowski, L. P., IPC – Isoelectric Point Calculator. *Biology Direct*. 2016, *11*, 55.

[2] Rabbani, N., Ashour, A., Thornalley, P. J., Mass spectrometric determination of early and advanced glycation in biology. *Glycoconjugate Journal*. 2016, *33*, 553-568.

[3] Efron, B., Better Bootstrap Confidence Intervals. *Journal of the American Statistical Association*. 1987, *82*, 171-185.
